# Decrypting molecular mechanism of heat stress tolerance in rice to tackle climate change challenges through recent approaches

**DOI:** 10.3389/fpls.2025.1722694

**Published:** 2026-02-05

**Authors:** Neeraj Kumar, Seyed Mahdi Hosseiniyan Khatibi, Deepak Sharma, Faraz Azeem, Ganesh Kumar Koutu, Jauhar Ali

**Affiliations:** 1 Rice Breeding Innovations Department, International Rice Research Institute, Los Baños, Laguna, Philippines; 2 Department of Genetics and Plant Breeding, Indira Gandhi Krishi Vishwavidyalaya, Raipur, Chhattisgarh, India; 3 Institute of Crop Science, College of Agriculture and Food Science, University of the Philippines Los Baños, Laguna, Philippines; 4 Directorate of Research and Extension Services, Jawaharlal Nehru Krishi Vishwavidyalaya, Jabalpur, Madhya Pradesh, India

**Keywords:** breeding, climate change, heat stress, microbiome, molecular response, rice, thermotolerance, high throughput phenotyping

## Abstract

Rice (*Oryza sativa*) is one of the world’s most important cereal crops, contributing to food and financial security, particularly in developing countries. High temperature due to climate change seriously threatens sustainable rice production. Rice crops are adversely affected by heat stress at the morphological, physiological, and molecular levels, resulting in reduced yield and poor grain quality. Rice is highly sensitive to heat during the reproductive phase, causing pollen sterility, impaired pollen dehiscence, pollen germination, and tube growth, ultimately drastically reducing spikelet sterility and yield. High temperature also promotes the accumulation of reactive oxygen species in plant cells, resulting in multiple adverse effects, including damage to chloroplasts and cell membranes, inactivation of photosystems, reduced Rubisco activity, and impaired production of photoassimilates. In this review, we have synthesized the current knowledge on the effects of heat stress on rice and summarized QTLs, genes, and regulatory pathways underlying thermotolerance. We further evaluate conventional breeding, transgenics, and diverse omics-based strategies to breed high-yielding, heat-tolerant rice varieties. The precise molecular insights gained through various omics approaches are expected to advance our understanding of the intricate nature of heat stress tolerance in rice. Additionally, we highlight the emerging roles of microbiome, high-throughput phenotyping technologies, and artificial intelligence as promising tools for accelerating the development of heat-resilient rice.

## 1 Introduction

**Figure 1:**
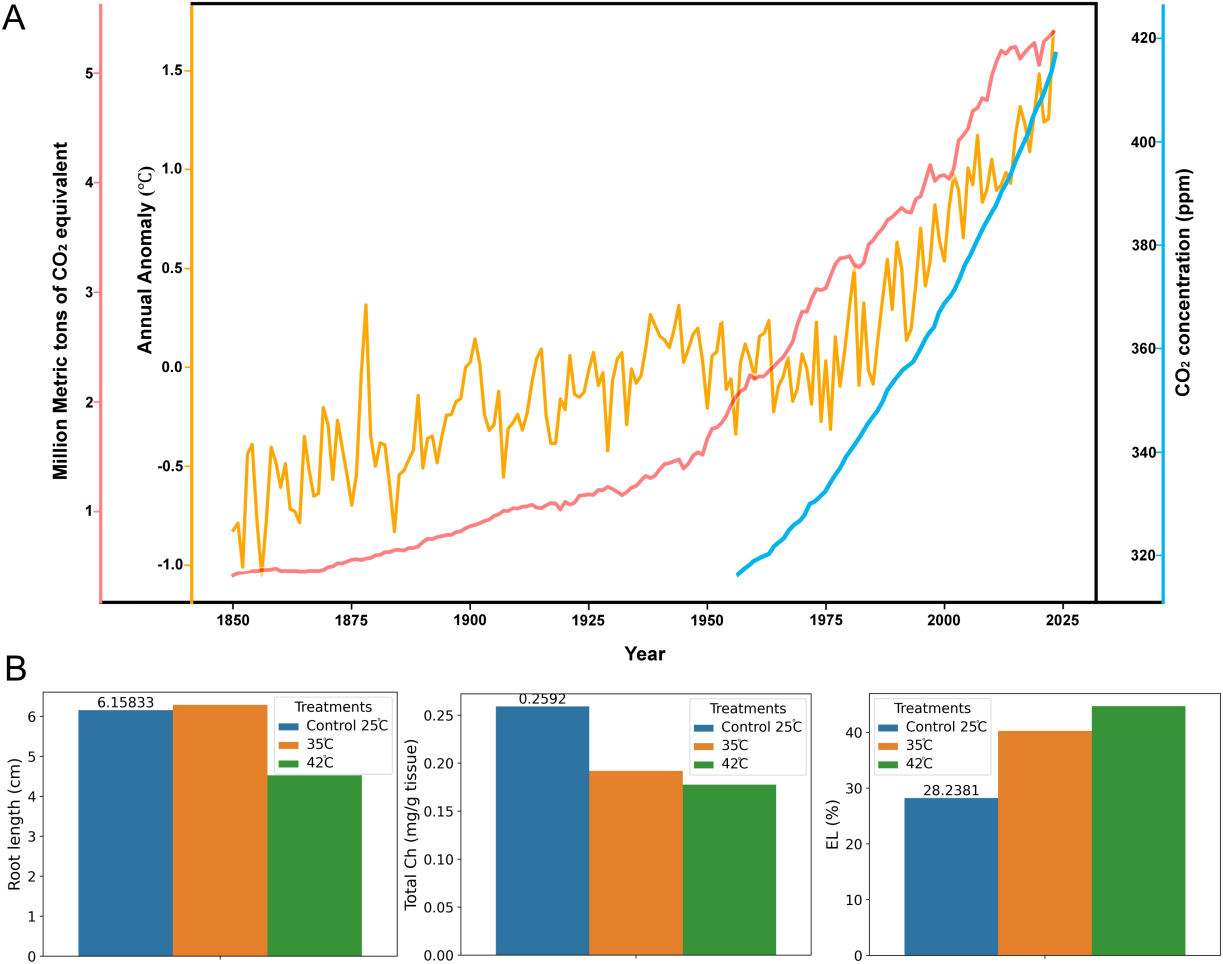
Global warming trends and impact of HS on some rice physiological traits **(A)** Annual global anomaly (°C) from 1850 to 2023 (Berkeley Earth, 2024), greenhouse gas (GHG) emissions (million metric tons of CO_2_ equivalent) (Jones et al., 2024 – with major processing by Our World in Data), and atmospheric CO_2_ concentration (ppm) from 1960 to 2023 (NOAA Global Monitoring Laboratory, 2024), illustrating global warming trends. GHGs, particularly CO_2_ emissions, are the primary drivers of increased mean air temperature; **(B)** Effects of HS treatments (control: 25°C, Moderate heat: 35°C, and severe heat: 42°C) on root length, total chlorophyll (Ch) content, and electrolyte leakage (EL) in rice, highlighting physiological responses to elevated temperatures. Increased EL at elevated temperatures indicates membrane damage (Taratima et al., 2022).

Rice (*Oryza sativa* L.), the most crucial staple food crop supporting over half of the global population, is cultivated across approximately 165 million hectares in 118 countries, with production exceeding 776 million tons in 2022 (FAOSTAT, 2023). Rice production is threatened by earth’s quickly changing ecosystems due to climate change. One of the significant abiotic stresses affecting rice production is high temperature (HT). Since 1850, global temperatures have increased by about 0.06°C per decade (**Figure 1A**), but warming rate has accelerated sharply to 0.20°C per decade since 1982, over three times faster (NOAA Climate, 2025). The roughly 1°C increase in global average surface temperature since the pre-industrial era (1850-1900), primarily due to greenhouse gas (GHG) emissions into the atmosphere by human activities, might seem small. Still, it means a significant increase in accumulated heat. Historically, the 10 warmest years have all occurred in the past decade (2014-2023). Remarkably, 2023 has been the warmest year by a wide margin. It was 1.18°C above the 20th-century average of 13.9°C. The extra heat leads to regional and seasonal temperature extremes (NOAA, 2024). In the near future (2021-2040), global warming will rise primarily because of the growing cumulative CO_2_ emissions in nearly all examined scenarios and modeled pathways. Global warming is more likely than not to reach 1.5°C in the near future even with very low GHG emissions, and is likely to exceed 1.5°C under moderate or high emission scenarios (Lee et al., 2023).

### 1.1 Rising global temperature and its effect on rice production

Prolonged exposure to temperatures surpassing a critical threshold (**Table 1**) conducive to optimal physiological functions inflict irreversible damage on plant growth (Khan et al., 2019). It is particularly significant in Southeast Asia, where rice plays a vital role, providing 3/4^th^ of the region’s calorific intake (Fitzgerald et al., 2009). However, this region is predicted to experience the most rapid temperature increase (IPCC, 2014). By 2030, it is extrapolated (Gourdji et al., 2013) that approximately 16% of the rice growing areas will encounter at least five reproductive days with temperatures surpassing Tcrit (physiological critical temperatures during the reproductive stage). This proportion is anticipated to rise to 27% by 2050. Between 2022 and 2023, a noticeable decline in global rice production, amounting to an estimated reduction of 300,000 tons compared to the preceding year, 2021-2022, was seen (USDA 2023). Notably, while specific rice-producing countries like India, Thailand, and Türkiye managed to sustain their production, several other prominent rice-producing countries have encountered considerable yield losses in recent years, attributed to the impacts of climate change. The trend is exacerbated by the fact that the world’s population is growing at a rate of 0.88% annually, necessitating expedited efforts to enhance rice productivity to keep pace with the demographic demand (UN DESA, 2022).

**Table 1:** Critical temperatures for the development of the rice plant at different growth stages.

Growth stages	Critical temperature		Adverse effects	Reference
	Optimum	High		
Germination	18-40	42-45	Poor germination rate reduced seedling vigor, altered enzymatic activity	(Liu et al., 2019)
Seedling emergence	25-30	35	Poor seedling growth, declined internal moisture levels, impaired metabolic rate, increased oxidative damage	(Bahuguna et al., 2015)
Rooting	25-28	35	Altered root system architecture, decreased root length, biomass, and nutrient uptake	(Taratima et al., 2022)
Leaf elongation	31	45	Decline in relative water content, photosynthetic and pigment concentration, increased evapotranspiration, wilting, curling, yellowing, and premature senescence of leaves	(Xu et al., 2020) (Kilasi et al., 2018)
Tillering	25-31	33	Reduced tiller numbers and biomass, effect on tiller angle	(Li et al., 2020)
Panicle Initiation	26.7-31.1	>31.1	Disruption of cell division and differentiation processes, smaller and fewer panicles	(Restrepo-Diaz and Garces, 2013; Sánchez et al., 2014)
Anthesis	30-33	35-36	Poor anthesis dehiscence, high spikelet sterility, distorted floral organs	(Arshad et al., 2017)
Pollination	25-35	_	Disrupted ion balance, carbohydrate metabolism, and phytohormones concentration in pollens, lessened swollen and poor pollen germination, reduced pollen number on stigma, abnormal pollen tube growth, and shortened stigma length	(Shrestha et al., 2022; Xu et al., 2020; Yoshida, 1981)
Ripening	20-29	>30	Shortened grain filling time, altered kernel size, reduced palatability, undesirable grain appearance, increased chalkiness, and decreased grain weight,	(Xu et al., 2021)

According to (Peng et al., 2004), research from the International Rice Research Institute (IRRI) during 1992–2003 indicates yield drop in rice by 10% for every 1°C rise in minimum temperature during the growing season. Similarly, a daytime temperature increase of 28°C to 34°C decreases the yield by up to 7-8% (Korres et al., 2017). A heatwave in Japan led to a 25% spikelet sterility rate when temperatures peaked around 38°C in 2007 (Hasegawa et al., 2011).

### 1.2 Projected declines in rice production due to elevated temperatures

Population growth has created a critical demand to ramp up crop production for food security. Estimates suggest that a 70% boost in food production will be vital to cater the demands of an anticipated 9 billion population by 2050 (Bita and Gerats, 2013). Short-term projections indicate that rice production in South Asia could decline by about 10% by 2030 (Lobell et al., 2008). Medium and long-term estimates predicts a 10-25% reduction in rice yields across developing countries by 2080, with India potentially facing losses of 30-40% due to extreme heat events (Cline, 2007). Overall, high temperature stress (HTS) may lower rice grain yield by up to 41% by the end of the 21^st^ century, as the temperatures are expected to rise by up to 2°C by 2050 relative to 1950 (Ceccarelli et al., 2010). Without the benefits of CO_2_ fertilization, effective adaptation measures, and genetic improvement, each 1°C rise in global average temperature is projected to decrease worldwide wheat yields by 6.0%, rice yields by 3.2%, maize yields by 7.4%, and soybean yields by 3.1% (Zhao et al., 2017). Spatial modeling predicts a 20% decrease in *boro* rice yield in Bangladesh by 2050, escalating to 50% by 2070, with average rice yields declining by up to 33% by 2081–2100 (Basak et al., 2009; Karim et al., 2012).

## 2 Heat stress combined with drought: amplifying stress responses in rice

Although rice is susceptible to heat and drought (Kumar et al., 2014; Venuprasad et al., 2007), the combination of drought and heat stress (HS) is the most common abiotic stress in field conditions, significantly impacting crop productivity. The simultaneous occurrence of drought and HS in various rice-growing regions is almost inevitable, leading to increased plant-tissue temperature as drought severity progresses. Mechanistic studies have shown that the combined exposure to drought and HS elicits a unique response rather than a simple additive effect of both stresses (Rizhsky et al., 2002; 2004). Despite recognizing the practical importance of combined drought and HS on plants, there is limited field-based knowledge in this area (Lawas et al., 2018). While the effects of combined heat and drought stress have been studied in model plants, relatively little information is available on rice’s response to these stresses, particularly during the critical flowering stage (Rang et al., 2011). Understanding the molecular mechanisms of tolerance to this stress combination during sensitive flowering and grain-filling stages in cereals, especially rice, remains limited (Lawas et al., 2019). Empirical screening for thermotolerance at different stages, and evaluating heat tolerance under combined stress conditions could accelerate the development of rice varieties with improved tolerance to multiple stresses (Costa et al., 2021).

### 2.1 Types of stress responses to heat in rice plants

Understanding the mechanisms by which rice plants respond to elevated temperatures is crucial for answering the key question: how do rice plants sense HT and then transduce signals into intracellular responses? This knowledge is equally critical to breeding rice cultivars with improved HS tolerance. Three types of different plant responses have been observed under HTS, namely basal thermotolerance, acquired thermotolerance (AT), and programmed cell death (PCD) (Guihur et al., 2021; Haider et al., 2021; Locato and Gara, 2018; Mittler et al., 2012). Basal thermotolerance is an inherent ability to survive HT above those conducive for growth and to acquire tolerance to lethal temperatures. In contrast, acquired thermotolerance, which is also known as adaptive thermotolerance, is the ability to withstand an otherwise lethal HT after being pre-exposed to a sublethal increased temperature, mimicking an ‘immunization’ against HT (Larkindale et al., 2005; Lim et al., 2013; Shanmugavadivel et al., 2019). Plants may remove some specific cells in response to HT or other environmental stimuli in a process called PCD (Locato and Gara, 2018).

## 3 Necessity for thermotolerance rice breeding

The need to breed heat-tolerant rice is crucial, considering its critical role in global food security and the detrimental effects of climate change on yield. To breed rice with heat tolerance, it is essential to elucidate the molecular basis of HS response in rice, the genes, proteins, and physiological and biochemical traits that confer heat tolerance (Janni et al., 2020; Raza et al., 2020; Sailaja et al., 2015). This review provides an overview of HS-induced morphological and physiological changes, elucidating molecular mechanisms underlying the HS response regulatory network in rice and strategies to breed for enhanced rice adaptation to global warming through various approaches.

## 4 Stage-specific effects of heat stress on rice

### 4.1 Effect of HTS on germination and vegetative growth

**Figure 2:**
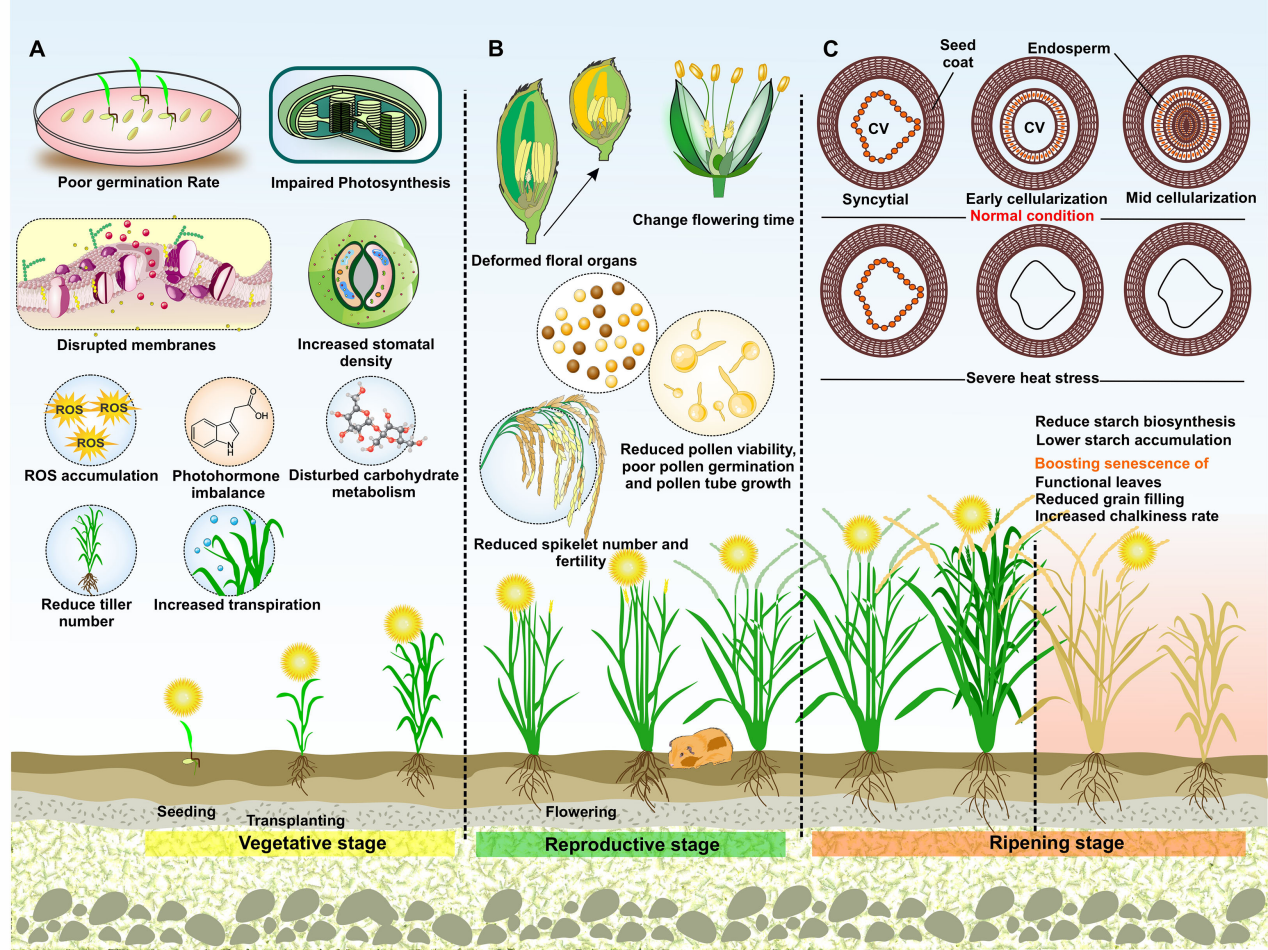
Effect of HS on rice at different stages. **(A)** Vegetative stage: HS during seed germination leads to low germination rates and week seedling vigor. Tiller numbers are reduced due to disrupted cell membranes, impaired photosynthesis, and accumulation of ROS. Phytohormone imbalances, increased water loss due to increased stomatal density, wilting, and impaired root growth further negatively impact the development of rice plants. **(B)** Reproductive stage: HS leads to altered flowering time, deformed floral organs, reduced pollen-viability, -germination and -tubegrowth, and spikelet sterility. Inhibited pollen dehiscence affects the amount of pollen landing on a stigma and negatively impacts fertilization rates. **(C)** Ripening stage: Decreased photosynthetic rate causes inhibited assimilate production and accelerates the senescence of functional leaves, resulting in reduced partitioning of carbohydrates into the grains. During the milky stage, HS hinders the synthesis and movement of carbohydrates, proteins, and lipids in developing grains. The initiation of endosperm cellularization is a critical developmental transition required for normal seed development. Impaired cellularization hinders endosperm development. Schematic drawing shows early stages of endosperm development (ED) under control and severe HS conditions. Under normal conditions, the rice seed development follows syncytial ED, early cellularization, and mid-cellularization stages. Under HS conditions, initiation of cellularization is severely affected. The central vacuole (CV) remains present when seeds are exposed to severe HS.

Each stage of rice plant development responds differently to HT (Zhang et al., 2018). HS has an impact on grain quality, dormancy, germination, and emergence in addition to seedling vigor and establishment across the entire seed development process (Brunel-Muguet et al., 2015; Finkelstein et al., 2008; Liu et al., 2019). Exposure to HT during seed germination leads to lower germination rates and decreased vigor in germinated seedlings (Fahad et al., 2017; Liu et al., 2019) (**Figure 2**). At the seedling stage, rice grows best at a temperature between 25 and 28°C. In seedlings, elevated temperatures (42–45°C) (**Table 1**) can damage cell membranes, hinder photosynthesis, and escalate oxidative damage, which results in increased water loss, wilting, impaired root growth (**Figure 1B**), and potentially plant death (Bahuguna et al., 2015; Liu et al., 2018). A decrease in germination and seed vigor due to HS has been associated with reduced plasma membrane (PM) thermostability and membrane fluidity (Fahad et al., 2017; Saidi et al., 2010). Lipid profiles of PM acclimatized to moderate HS revealed a marked reduction in fatty acid unsaturation, leading to increased membrane rigidity. This structural change accounts for the attenuated Ca^2+^ influx observed during HS (Saidi et al., 2010; Sangwan et al., 2002). Begcy et al. (2018) reported that HS (35°C) dramatically decreases the size of grain at maturity because of lower length, breadth, and mature grain weight during early grain development; and when the temperature reaches 39°C, the endosperm collapses, and seed viability is significantly reduced. Tillering, a crucial agronomic trait in rice, is severely affected by HT and thus reduces the number of panicles per plant. Soda observed a 35% reduction in panicle number and a 28% decrease in yield per plant in rice plants exposed to elevated temperatures (Soda et al., 2018). Other morphological traits to assess under HS include leaf drooping and rolling, reductions in plant biomass, and decreased chlorophyll concentration (Ali et al., 2022; Ren et al., 2023; Sita et al., 2017).

Rice seedlings’ ability to withstand HT varies depending on their genetic composition. The domestication origins of the two subspecies, *japonica* and *indica*, differ. The *japonica* emerged in the temperate regions, while the *indica* originated in tropical areas. The *indica* exhibits greater thermotolerance than *japonica* and possesses distinct morphological and physiological traits (Lee, 2002; Lee et al., 2017). HS affects tiller and panicle numbers more significantly in *Japonica* rice relative to *Indica* rice (Wang et al., 2016). Regarding heat resistance, hybrid rice varieties combining *indica* and *japonica* demonstrate the highest level, followed by *indica* and then *japonica* varieties individually (Prasanth et al., 2017).

### 4.2 Impact of HTS on the reproductive stage

**Figure 3:**
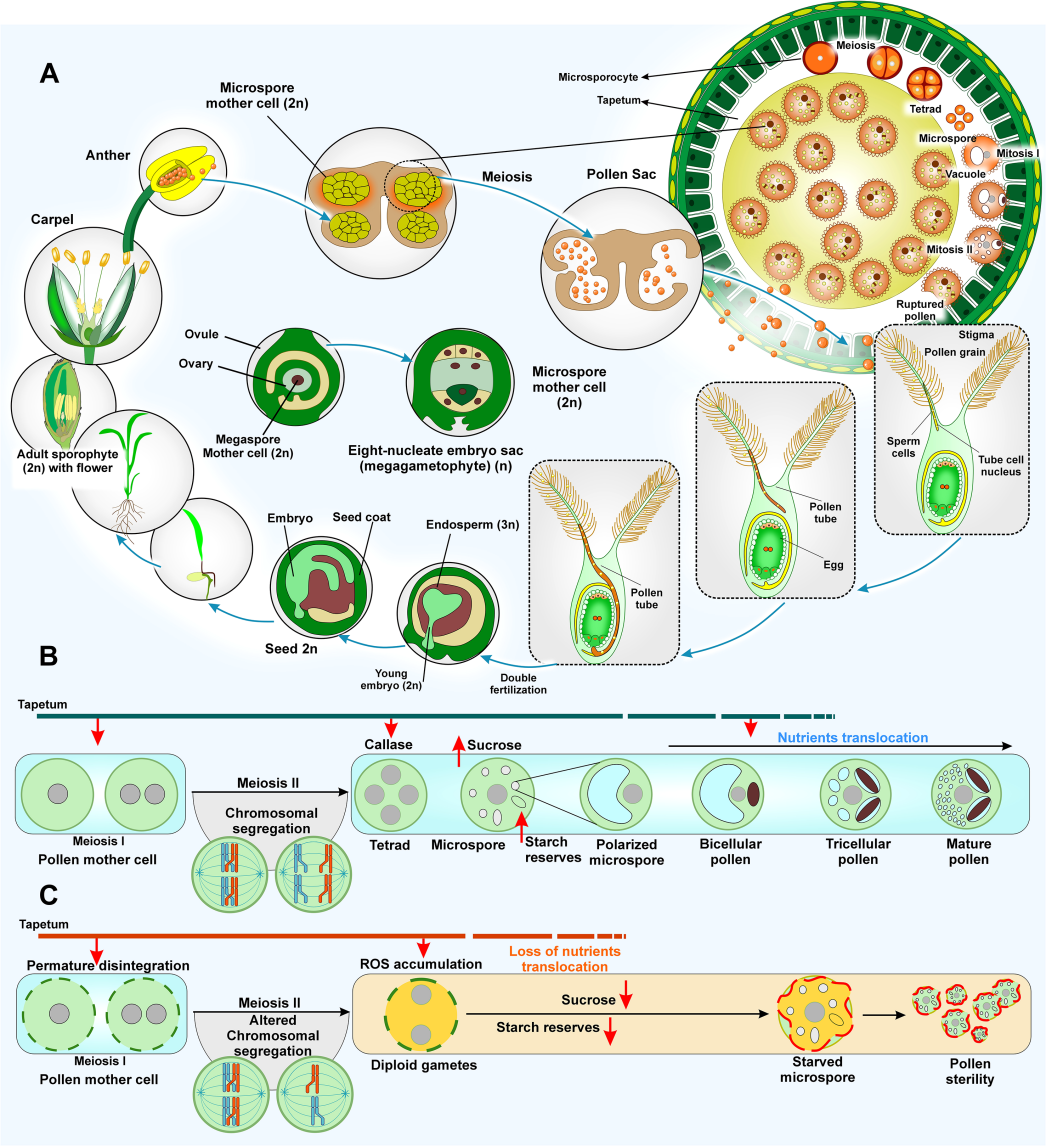
Impact of HS on the reproductive phase. **(A)** Impaired male and female gametophyte development: Floral organs are deformed under HS and have reduced numbers and sizes. HS leads to significant changes in gene expression, resulting in tapetum degeneration and pollen sterility in developing anthers during the early microspore stage. Swelling of pollen grains is restricted at the time of heading under HS conditions. Pollen fertility also decreases due to delays in the opening of the locule. HS severely reduces anther dehiscence during anthesis. Pollen viability and germination are reduced due to decreased protein and iron content in pollen grains. Pollen tube growth is significantly affected by HS. **(B)** Male gametophyte development under normal conditions. **(C)** Male gametophyte development under HS conditions: Developing pollen and the surrounding tapetal cells are highly sensitive to HS, resulting in premature degeneration of tapetal cells, causing disruptions in the supply of nutrients to developing pollens. HS affects the meiotic cell division by influencing the orientation of the spindle apparatus, resulting in aberrant chromosome behavior and failure of pollen development. ROS accumulation is increased, and soluble carbohydrate and starch reserves are decreased in developing anthers under HS, leading to starved microspores and increased pollen sterility.

The reproductive phase (panicle initiation to physiological grain maturity) is the most vulnerable stage to abiotic stresses (Guan-fu et al., 2008). The stages of panicle initiation, formation of male and female gametophytes, anthesis, pollination, and fertilization are most vulnerable to HS (Arshad et al., 2017; Jagadish et al., 2015). According to Xu et al. (2020), both daytime and nighttime HS causes deformation of floral organs reducing their size and number. During anthesis, HS impairs pollination, significantly increasing spikelet sterility (Sarangthem et al., 2021). Reactive oxygen species (ROS) is accumulated in plant cells during HT, resulting in multiple adverse effects such as damage to the chloroplast and cell membranes, loss of activity of photosystems, suppressed RuBisCo activity, and decreased production of photoassimilates. These issues culminate in poor flowering and decreased grain yield (Lal et al., 2022; Radha et al., 2022; Zaidi et al., 2019). HT has multiple adverse effects on rice stamens as (I) elevated temperatures disrupt meiosis during the pollen development, disintegration of tapetum and/or reduced activity of invertase enzyme, leading to the production of sterile pollen (Endo et al., 2009) (**Figure 3**), (II) HT inhibit pollen dehiscence and reduce the swelling capacity of pollen grains, which diminishes pollen amount landing on a stigma and negatively impacts fertilization rates (Arshad et al., 2017; Hu et al., 2021), (III) the moisture content adjusts of the pollen grains is essential for their formation and dispersion. The pollens landing on stigma adjust their moisture levels to environmental conditions, but HT can disrupt this process (Das et al., 2014; Shrestha et al., 2022), (IV) HT significantly decreases the protein content in the pollen, decreasing its germination ability and pollen-tube elongation rates, which ultimately leads to spikelet sterility (Arshad et al., 2017; Jagadish, 2020; Shrestha et al., 2022). HTS during anther formation, particularly during pollen mother cell meiosis, can lead to early deterioration and breakup of tapetal cells. This affects the nutrition of microspores and the generation of pollen walls, culminating in abortion of pollen grains and reduced stigma length (Liu et al., 2020; Xu et al., 2020; Zhang et al., 2018) (**Figure 3**). An *indica* variety, IR64, showed 66% reduction in the number of spikelets when it was exposed to HS (40°C day/35°C night) at pre-flowering stage to HS conditions for 15 days (Soda et al., 2018). Hu et al. (2021) observed a decline in pollen viability, spikelet fertility, and grain yield by 46%, 69%, and 84%, respectively under HS in a heat susceptible variety Liangyoupeijiu (LYPJ) in comparison to 18% yield reduction in Shanyou63 (SY63), a heat tolerant variety. Lin et al. (2023) found that heat-sensitive rice mutant, *heat shock protein60-3b (oshsp60-3b)*, showed decreasing fertility as temperature increases. Overexpression of *OsHSP60-3B* enhanced thermotolerance of pollen in transgenic plants. Multiple genes regulating heading in rice such as *Hd1* (heading date 1), *Ehd1* (early heading date 1), *Ghd7* (grain number, plant height, and heading date 7), and *Hd3a/RFT1* (heading date 3a/rice flowering locus T1) form the core Ghd7-Ehd1-*Hd3a/RFT1* flowering pathway. HT reduces *GhD7* transcript levels, reducing its inhibitory effect on RFT1 and enabling timely floral induction. A natural allele of qHd1 (encoding OsMADS51) further enhances heat tolerance at heading and grain filling through OsMADS51-Ehd1-Hd31/RFT1 pathway (Kan et al., 2023; Zhou et al., 2021; Xue et al., 2008).

**Figure 4:**
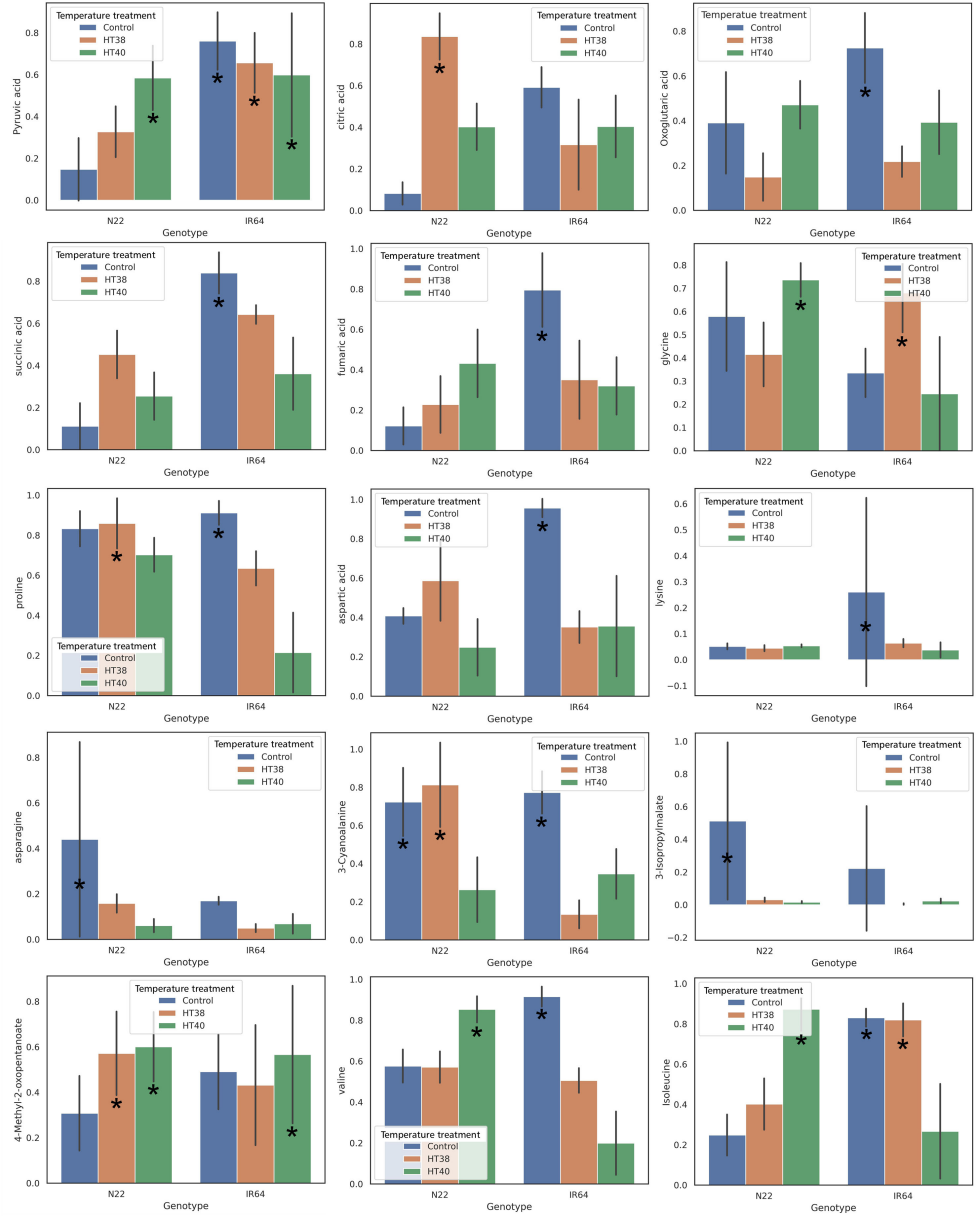
Metabolic profiling of pistils at the end of HS in N22 and IR64. Metabolite profiling of pistils in varieties N22 (heat tolerant) and IR64 (heat sensitive) after HS temperature treatment reveals the distributions of metabolites under control (30°C) and HS conditions (38°C and 40°C). Metabolites involved in significantly altered metabolic pathways are shown here. Under control conditions, IR64 showed higher concentrations of most of the metabolites compared to N22, except for glycine and asparagine, which were significantly lower in IR64. After HS treatment in N22, all the metabolites from the TCA cycle (pyruvic acid, citric acid, oxoglutaric acid, succinic acid, fumaric acid) increased under 38 °C and 40 °C compared to the control, except for oxoglutaric acid at 38 °C (Shi et al., 2022). "*" represents significant difference between genotype metabolites for different temperature treatments.

In addition, HT severely impact the further fertilization processes. Restricted pollen tube growth hinders the pollen movement towards egg cells because of disruption in the ion balance, carbohydrate metabolism, and phytohormone concentration of pollens (Coast et al., 2016; Firon et al., 2006; Jagadish, 2020; Yan et al., 2002). Following double fertilization, a short term exposure to HTS (39°C for 48 hours) leads to impaired cellularization during initial endosperm development, hindering the subsequent establishment of the endosperm (Folsom et al., 2014). Many studies in recent years have been carried out to investigate the effect of HS on the morphology of the reproductive parts. Still, fewer reports are available on the impact of HS on stigma. Jagadish et al. (2010) observed reduced stigma length when they exposed the rice plants to HT for 6 hours during anthesis. Increased stigma length may enhance tolerance to HS during the flowering period. Callose could be used as an indicator of sterile ovules, with its deposition at the ovule chalaza commonly used to assess early ovule degeneration (Endo et al., 2009). Evaluating and selecting rice varieties on the basis of characteristics such as rapid pollen dehiscence (Kobayashi et al., 2011), proper septum breakage during pollen expansion (Matsui and Omasa, 2002), and increased pollen protein content (Arshad et al., 2017) can provide more comprehensive insights into pollen quality and offer superior indicators than pollen viability alone when screening for HS tolerance. Additionally varieties with enhanced internal anthocyanin concentration have been shown to protect the photosynthetic apparatus via ROS scavenging mechanism, thereby improving thermotolerance (Zaidi et al., 2019). Early-flowering rice varieties, which can escape HTS, are also considered cost-effective and widely adopted in plant breeding. The QTL *qEMF3*, detected in *Oryza officinalis*, shifts the flower opening time of cultivars to earlier in the morning (Hirabayashi et al., 2015; Jagadish, 2020; Jagadish et al., 2007). Substantial changes in the metabolic profiles of different tissues in rice are observed under HS conditions. Specifically, heat-tolerant rice varieties exhibit a unique build-up of crucial metabolites, setting them apart from heat-sensitive types (Singh et al., 2024) (**Figure 4**). Consequently, analyzing the morphology and physiology of flowers in various heat-tolerant and -sensitive varieties could assist plant breeders in identifying and selecting for thermotolerance.

### 4.3 Impact of HTS on rice grain development and quality

**Figure 5:**
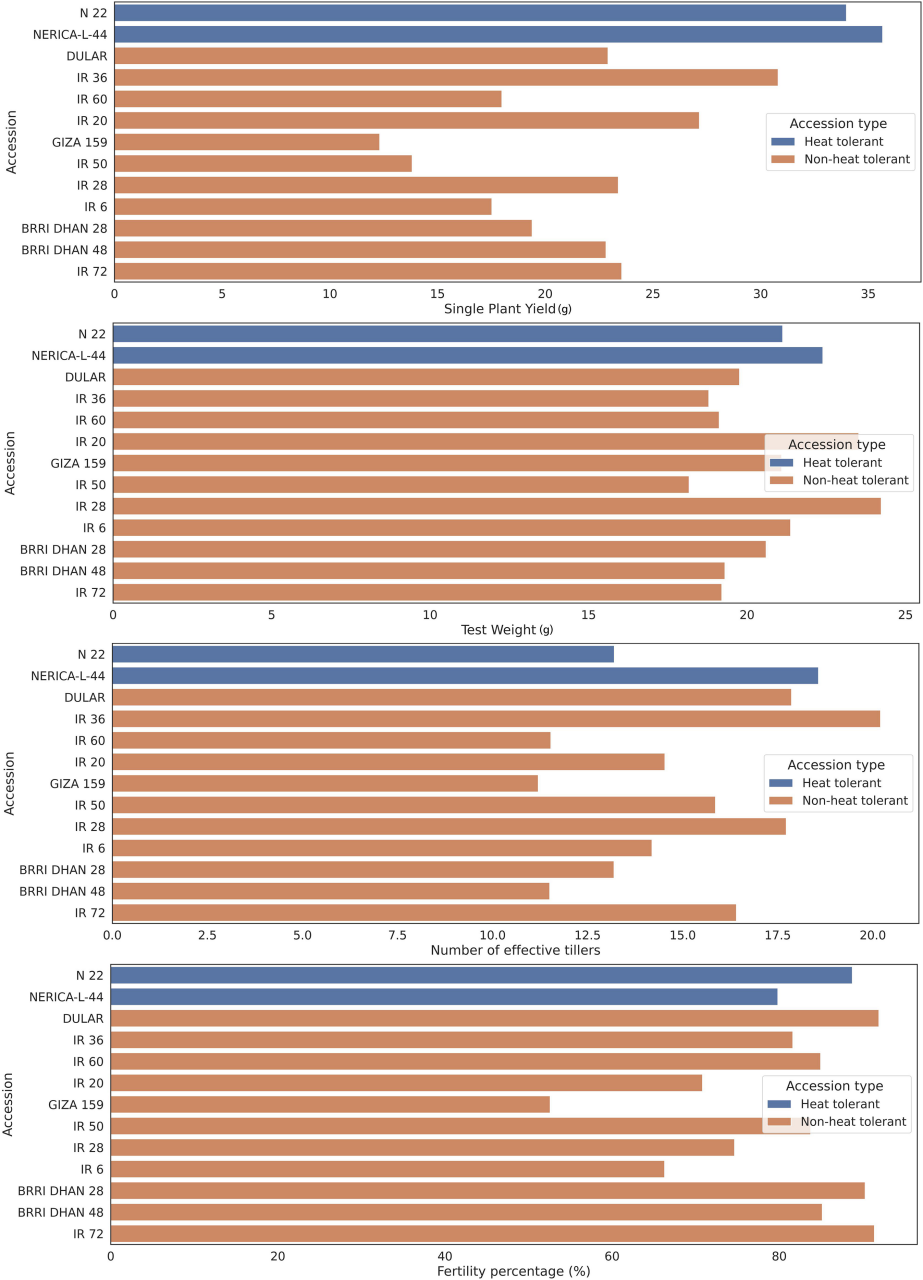
Impact of HS on different yield parameters of various rice varieties. Different popular rice varieties show a wide range of variation compared with heat-tolerant varieties (N22 and NERICA-L-44) under HS conditions for single plant yield (grams), test weight (grams), number of effective tillers, and spikelet fertility percentage (Surender et al., 2021).

Elevated temperatures hinder the transport and biosynthesis of sugars, proteins, and lipids in rice caryopses during the milky stage (**Figure 2**), consequently affecting grain weight and quality (Liao et al., 2012; Sreenivasulu et al., 2015; Zhang et al., 2016). An enhanced rate of grain filling and a reduced total grain filling duration of 21.3%–37.1% for various genotypes after HS treatment at the grain-filling stage have been reported. After being subjected to HT (38 °C/30 °C day/night) continuously for 20 days at grain filling stage, the seed weight decreased by 24.6% for tolerant Nagina 22 (N22) and 39.1% for sensitive IR64 compared to normal conditions (31 °C/23 °C day/night) (Shi et al., 2017; Zhang et al., 2022). Grain filling is more prone to high night temperature (HNT) stress (Krishnan et al., 2011). HT inhibits assimilate synthesis by decreasing photosynthesis (Zhang et al., 2009) and accelerating the senescence of functional leaves, resulting in reduced assimilate transfer to grains. Additionally, elevated temperatures may hinder early embryo (Cao et al., 2016) and seed formation (Huang et al., 2019). HT can supress gene expression and often disrupt the bioactivity of starch-producing enzymes, thus disturbing starch accumulation and the ratio of amylose to amylopectin in the endosperm of rice (Chen et al., 2017; Impa et al., 2021; Yamakawa and Hakata, 2010; Zhang et al., 2021). HS has also resulted in lower grain weight, decreased grain width, altered kernel size, and reduced yield (Folsom et al., 2014; Lyman et al., 2013; Rangappa et al., 2024; Shi et al., 2017) (**Figure 5**). HT at grain filling stage has shown to cause a significant decrease in rice yield, with losses of up to 50% (Sreenivasulu et al., 2015). Under HS conditions, a decrease of 16.7% in number of grains per plant led to a significant reduction of 18% in grain yield at HNT (28°C from 6 pm to 6 am) (Sharma et al., 2024).

In addition to affecting yield, rice grain quality is also compromised. It has been demonstrated that HT during the grain filling period increases the chalkiness rate and decreases head rice rate, gel consistency, amylose content, and overall taste quality (Dou et al., 2024). Setback viscosity, average particle size, crystallinity, and gelatinization temperature increased when the plants were subjected to 34°C (5°C higher than the normal temperature) (Zang et al., 2022). HTS accelerate non-uniform grain filling due to rapid endosperm cell division, ultimately shortening the filling period. Poor starch accumulation pattern and loose packing of starch granules results in chalkiness, brittleness, fissured, and broken grains. These reduces palatability, appearance, and milling quality of rice (Sreenivasulu et al., 2015; Yao et al., 2020; Shirdelmoghanloo et al., 2022; Zhang et al., 2021; Nevame et al., 2018). When plants were subjected to HS during the grain filling stage at 38/30°C (day/night) temperature, increased chalkiness (averaging 196.6%), reduced length/width ratio, increase in protein content (7.8 – 29%), decrease in total starch content (1.5%), decrease in amylose (6 - 11.5%) and decrease in amylopectin (5.2%) were observed (Zhang et al., 2023).

Starch in rice grains is the primary reserve, and its biosynthesis is very sensitive to heat. Among the various enzymes contributing to starch biosynthesis, ADP-glucose pyrophosphorylase (AGPase) is particularly susceptible to HS in the seed maturation phase. To increase thermotolerance for this enzyme in rice, Hwang et al. (2019), manipulated two dominant subunits of AGPase present in the developing endosperm, the large (L2) and small (S2b) subunits of cytosol-specific AGPase.

## 5 Impact of HTS on rice physiology

### 5.1 Impact of HTS on membranes

Plant cells’ primary protective barriers are biomembranes, composed of highly organized lipids and proteins, that are extremely sensitive to heat (Niu and Xiang, 2018; Sita et al., 2017). Impaired plant growth and development are often associated with plant cells’ disrupted physiological and metabolic processes. The increased kinetic energy and protein mobility stimulated by elevated temperatures lead to molecular bonds within membranes (Dhanda and Munjal, 2012). HST can disrupt the composition and function of PM, altering the ratio of saturated to unsaturated fatty acids and inducing protein denaturation. This disruption leads to elevated fluidity and permeability, impaired membrane integrity, and higher leakage of cells’ ions (Xalxo et al., 2020). The primary response to elevated temperatures also occurs in the PM, which activates the channels and receptors essential for HS sensing, cellular response, and transduction of calcium signalling. HTS also hampers the activity of fatty acid desaturases, hence affecting the extent of unsaturation of fatty acid chains, crucial for HS adaptation in plant (Higashi and Saito, 2019; Lamers et al., 2020; Niu and Xiang, 2018; Shen et al., 2015).

To survive extreme temperatures, plant cells must first ensure the stability and fluidity of their lipid membranes by modulating lipid saturation, with fatty acids being the fundamental components. *HTS1* is crucial for *de novo* fatty acid biosynthesis, and its scarcity inhibits fatty acid synthesis and metabolism of fats in *hts1* mutants. This reduction in fatty acid level compromises cell membrane’s integrity and stability under HS, which leads to abnormal heat-induced calcium signaling (Chen et al., 2021). *OsCNGC14* and *OsCNGC16*, which are cyclic nucleotide-gated ion channels located in the PM, regulate calcium signals in response to HTS, thereby conferring thermotolerance to rice (Cui et al., 2020).

Membrane thermostability is the most reliable trait for screening heat-tolerant rice genotypes, showing a strong correlation with yield under HT. Mean relative injury and leaf electrolyte leakage are increased by HTS (**Figure 1B**). Therefore, genotypes with high membrane thermostability and low relative injury are promising candidates for direct selection or hybridization in future breeding programs for rice thermotolerance (Sailaja et al., 2015; Maavimani et al., 2014).

### 5.2 HTS induced alterations in photosynthesis

HS disrupts the permeability of the thylakoid membrane and even cause the disintegration of thylakoid grana, which leads reduced in chlorophyll (**Figure 1B**) and altering photochemical reactions, causing a reduction in ratio of variable fluorescence to maximum fluorescence (Fv/Fm) and rate of photosynthesis (Chakraborty and Bhattacharjee, 2015; Hu et al., 2020; Wang et al., 2018). Among these components, photosystem II (PS II) is particularly susceptible to HS, with oxidative stress induced by the HT causing the dissociation of the oxygen-evolving complex (OEC) in PSII, thereby restricting electron transport from OEC towards the acceptor side of PSII, thus causing a substantial decline or complete loss in its activity (Essemine et al., 2017; Sailaja et al., 2015; Szymańska et al., 2017). Moreover, HT inhibits the activity of ribulose-1,5-bisphosphate carboxylase/oxygenase (RuBisCo), primarily due to the inactivation of RuBisCo activase (Perdomo et al., 2017). Other than this, HT also reduces amounts of photosynthetic pigments and leads to reduced carbon fixation potential (Hasanuzzaman et al., 2013; Song et al., 2014).

Genetically engineered transgenic plants having increased levels of RuBisCo activase showed improved growth in HT conditions and demonstrated higher photosynthetic rates compared to wild-type plants (Wang et al., 2010). Overexpression of a thermotolerant RuBisCo activase from wild rice significantly enhanced growth and grain yield of cultivated rice during HT, indicating that manipulating RuBisCo activase could be an efficient strategy for thermotolerance rice breeding (Scafaro et al., 2018). To prevent damage buildup, PSII plants utilize the *de novo* synthesis of proteins, including the D1 subunit protein (core subunit of PSII, susceptible to light and HS) encoded by the chloroplast gene *psbA*, which is crucial for the process. Introducing a heat-responsive promoter to enhance D1 protein expression increased heat tolerance, resulting in significant increases in both aboveground biomass (20.6%–22.9%) and grain yield per plant (8.1%–21.0%) compared to regular rice plants (Chen et al., 2020). The absence of *OsNSUN2* (an RNA 5-methylcytosine (m^5^C) methyltransferase) function results in a weakened photosystem characterized by decreased efficiency in photosynthesis and the accumulation of ROS following exposure to heat (Tang et al., 2020). Ahmad et al. (2024) observed that the gene *PALE GREEN LEAF 10 (PGL10)* is essential for chlorophyll synthesis in rice. Loss of *PGL10* function results in pale green leaves and impaired photosynthesis under HT conditions. Thus, thoroughly understanding how photosynthetic metabolism responds to HS is essential for examining plant resilience and recognizing the detrimental effects of HT on agricultural productivity (Bita and Gerats, 2013).

### 5.3 Impact of HTS on carbohydrate metabolism and partitioning

HTS alters carbohydrate metabolism and the distribution of photo-assimilates in rice plants (Arshad et al., 2017; Bahuguna et al., 2016; Shi et al., 2017). The levels of two crucial enzymes involved in the glycolytic pathway, phosphoglucose isomerase and phosphofructokinase, were significantly reduced, along with a decrease in the abundance of phosphoglycerate mutase, when rice cells were subjected to HT (44°C). This suggests that a cell’s ability to produce energy under HS is hampered (Gammulla et al., 2010). Compared to wild-type plants, a heat-resistant rice mutant, *ett1*, demonstrated increased survival, less oxidative damage, and higher photosynthetic efficiency under HTS. This mutant’s accumulation of higher energy and carbohydrates suggests enhanced metabolic activity and stress tolerance (Feng et al., 2023). A transcription factor (TF), HYR (higher yield rice) involved in carbon metabolism, enhances photosynthesis under HS (Ambavaram et al., 2014).

HTS disturbs sugar content in anthers, disrupting the regular nutrient supply essential for developing pollens (De Storme and Geelen, 2014; Rezaul et al., 2019). In rice varieties sensitive to heat, the Carbon-Starved Anthers (*CSA*) gene has increased expression. In contrast, heat-resistant varieties show robust expression of the sugar transporter gene *MST8* and the cell wall invertase gene *INV4*. This suggests that sugar deficiency has a significant function in spikelet sterility (Li et al., 2015). In a similar vein, compared to a sensitive cultivar, the thermotolerant rice cultivar expressed more of the sucrose transporter gene *OsSUT1*, which increased the amount of photo-assimilates available for filling kernels (Miyazaki et al., 2013).

### 5.4 Impact of HTS on phytohormones

Plant hormones are vital for regulating growth and development of rice plants under optimal and adverse environments by triggering many signaling cascades to facilitate the adaptive responses of plant (Khan et al., 2023). Exogenous application of various compounds, including antioxidants (ascorbic acid, alpha-tocopherol), amino acids (glycine betaine), and plant hormones (salicylic acid, auxins, brassinosteroids, methyl jasmonates) has highlighted the significant role of phytohormones in mitigating the adverse effects of HTS on rice (Fahad et al., 2016; Mohammed and Tarpley, 2011). During HTS, salicylic acid has been found to reduce the accumulation of ROS in anthers to mitigate pollen abortion by preventing premature degradation resulting from tapetum programmed cell death (Feng et al., 2018; Nadarajah et al., 2021). Melatonin deficient rice mutants coupled with reduced brassinosteroids (BRs) synthesis exhibited enhanced tolerance to HS (Hwang and Back, 2019). Ethylene-mediated signaling pathways aid in minimizing oxidative damage, preserving chlorophyll content, and regulating metabolism of carbohydrates which leads to improved thermotolerance in seedlings (Gautam et al., 2022). HS alters the balance of phytohormones, causing decreased active cytokinin (CTK), gibberellin (GA), and indole-3-acetic acid (IAA) contents in rice spikelets and developing kernels. This disruption hinders cell proliferation and panicle formation, decreasing spikelet number, pollen fertility, and kernel weight. Conversely, HS results in elevated abscisic acid (ABA) content in anthers and seeds, which, as a result, induces pollen abortion and inhibits germination and seedling establishment (Liu et al., 2019; Tang et al., 2008; Wu et al., 2016). Exposure to HS inhibits CTK transportation rate and CTK synthesis enzymes while increasing cytokinin oxidase/dehydrogenase activity, particularly in heat-sensitive cultivars. These changes likely contribute to decreased panicle CTK abundance under HS conditions (Wu et al., 2017).

Zhang et al. (2018) studied how levels of naturally occurring ascorbic acid affect the use of transgenic rice plants. These plants either had increased or decreased activity of L-galactono-1,4-lactono dehydrogenase (GLDH) enzyme, which catalyzes the terminal step in ascorbic acid biosynthesis pathway. When GLDH activity was suppressed, higher levels of ROS were observed in the transgenic rice. However, when endogenous ascorbic acid was present at higher levels, it inhibited the breakdown of RuBisCo and chlorophyll. Consequently, it reduced ROS accumulation, enhancing rice plants’ stability when exposed to HT. *OsNCED1 (9 CIS-EPOXYCAROTENOID DIOXYGENASE)* can control the endogenous ABA content in rice. This enzyme enhances the antioxidant capacity, activates the expression of gene associated with heat and ABA, and positively regulates rice seedling thermotolerance (Zhang et al., 2022). By upregulating two heat-responsive genes, *JASMONATE ZIM DOMAIN (JAZ)*, and the heat tolerance gene on chromosome 3 (HTG3) controls rice thermotolerance (Wu et al., 2022).

Thus, comprehending how these plant hormones function will significantly aid in identifying the mechanisms of HT tolerance in rice plants. To mitigate the impact of HS on yield losses, strategies could involve manipulating phytohormone signaling pathways to develop varieties with enhanced heat tolerance.

### 5.5 Impact of HTS on ROS accumulation

Studies have shown that the exposure to HTS induces a rapid ROS burst in plant tissues and disrupts the homeostasis between ROS production and detoxification (Baxter et al., 2014; Huang et al., 2016; Zhao et al., 2023). ROS affects the redox homeostasis and the function of proteins including their transcriptional activities and enzymatic properties during stress situations (Mittler, 2017). HT triggers the production of hydrogen peroxide (H_2_O_2_) primarily in chloroplasts and mitochondria. This compound not only serves as an early messenger in cellular signaling but also inflicts damage on cells over time, potentially leading to programmed cell death (Qiao et al., 2015), growth retardation, and grain chalkiness (Suriyasak et al., 2017), seedling death (Fang et al., 2015) and spikelet sterility. In rice plants experiencing HTS (with days at 38 °C and nights at 30°C during meiosis), the ROS concentration in anthers exceeds threefold that of normal temperature conditions (with days at 28 °C and nights at 22°C). HTS also induces a surge in ROS levels in rice pistils, likely due to the upregulation of Respiratory Burst Oxidase Homolog (RBOH) genes (Fu et al., 2016; Zhang et al., 2018, 2023). Additionally, HTS hampers the activity of antioxidant enzymes, particularly superoxide dismutase (SOD) and catalase (CAT) (Sailaja et al., 2015; Zhang et al., 2018).

*OsANN1*, an annexin that binds calcium, enhances thermotolerance through modulating antioxidants accumulation such as CAT and SOD under HT (Qiao et al., 2015). Excessive ROS, particularly, exacerbates membrane lipid peroxidation and protein oxidation, resulting in heightened levels of intracellular malondialdehyde (MDA), which can disrupt the normal functioning of proteins and nucleic acids (Bahuguna et al., 2015; Chakraborty and Bhattacharjee, 2015). Therefore, indicators such as electrolyte leakage, ROS levels, expression levels of antioxidative genes, activities of antioxidant enzymes, and MDA content are regularly employed to assess membrane and oxidative damage and measure plants’ heat tolerance. For instance, heat-tolerant rice varieties like NERICA-L-44 and N22 demonstrate increased membrane stability and reduced ROS and MDA levels due to elevated antioxidant enzyme activities (Bahuguna et al., 2015; Higashi and Saito, 2019; Sailaja et al., 2015). Heat-sensitive mutants accumulated ROS, reduced catalase activity, and upregulated *OsSRFP1* (a RING finger E3 ubiquitin ligase) expression under HS (Zafar et al., 2020). Overexpressing *OsProDH* (encoding protein dehydrogenase) lines had reduced proline content while its knockout mutant lines had enhanced proline content. Increased proline content reduced H_2_O_2_ accumulation in the seedlings of mutant lines indicating *OSProDH* regulating HS tolerance negatively (Guo et al., 2020).

Monodehydroascorbate reductase (MDHAR), encoded by *OsMDHAR4*, is a scavenger of ROS. *OsMDHAR4* negatively impacts rice thermotolerance by modulating stomatal responses induced by H_2_O_2_. Inhibition of *OsMDHAR4* promotes stomatal closure, increases H_2_O_2_ accumulation, reduces water loss, and enhances heat tolerance (Liu et al., 2018). The pyridoxal phosphate homeostasis protein (PLPHP), encoded by *HTH5* and mitochondria-localized, may mitigate damage to mitochondrial energy metabolism during HT by controlling ROS dynamics. Overexpressing *HTH5* significantly mitigates ROS accumulation triggered by HS (Cao et al., 2022).

## 6 Impact of HTS on protein homeostasis

Another significant consequence of HS is the perturbation of protein homeostasis or proteostasis within cells, resulting in cell death and toxicity. The term proteostasis includes processes associated with biogenesis, folding, unfolding, trafficking, and turnover of proteins (Mishra and Grover, 2016). HS often causes protein misfolding, unfolding, and protein denaturation or aggregation in the endoplasmic reticulum (ER) and triggers the unfolded protein response (UPR) to restore ER protein homeostasis (Liu and Howell, 2016; Sun et al., 2021; Zhang et al., 2017). A group of specialized proteins known as Heat Shock Proteins (HSPs) function as chaperones and can be crucial in stabilizing, correcting, refolding, restructuring, compartmentalizing, or breaking down misfolded proteins (Sharma et al., 2019). *OsHSP101* has been observed to perform a key function in enhancing the long-term acquired thermotolerance in rice by forming a positive feedback loop with *HSA32(HEAT STRESS ASSOCIATED 32-KD PROTEIN)* (Lin et al., 2014). When toxic proteins accumulate rapidly, proteasome-mediated degradation proves more effective than HSPs in restoring the denatured proteins. Specifically, *TT1* (*Thermotolerance1*), an α2 subunit of the 26S proteasome, efficiently removes cytotoxic denatured proteins associated with ubiquitination, helping to maintain protein homeostasis during HS. Overexpression of *OgTT1* markedly increased thermotolerance in rice, *Arabidopsis*, and *Festuca elata* (Kan and Lin, 2021; Li et al., 2015).

## 7 Impact of HTS on source and sink dynamics

During HTS plants experience cellular disruption due to redox imbalance and increased ROS leading to reduced photosynthetic efficiency and impaired activities of enzymes associated with sugar metabolism, ultimately affecting source-sink dynamics (Gautam et al., 2022; Hassan et al., 2020). Grain filling consists of using carbohydrates like sucrose, made in the leaves, i.e., source. These sugars travel long distances through the phloem, which acts as a transport pathway, and are finally deposited into the developing endosperm, where they are stored as starch granules in amyloplasts (Nazir et al., 2023; Ren et al., 2023).

*GRAIN FILLING RATE 1 (GFR1)* boosts sucrose production in leaves by enhancing RuBisCo activity, consequently regulating the grain filling rate (Liu et al., 2019). Sucrose is primarily transported from source to sink tissues via sieve elements (SEs) (Xu et al., 2017). Overexpressed *OsNAC23* gene enhances sugar transport to sink organs and lowers levels of sucrose, nonstructural carbohydrate, and temporary starch accumulation. In contrast, its absence has the opposite effect in mutants (Li et al., 2022). Shi et al. (2013) evaluated nitrogen (N) and nonstructural carbohydrate translocation into grains, impacting yield, grain-filling dynamics, and quality in N22 and Gharib (heat-sensitive) under HNT. Reduced grain yield in Gharib resulted from decreased N and nonstructural carbohydrate translocation post-flowering, affecting grain-filling rate, weight, and quality. Enhanced HNT tolerance in N22 correlated with increased HSPs and calcium-signaling proteins, along with efficient protein modification and repair mechanisms, particularly during early grain-filling. Accelerated grain-filling rate and improved proteomic protection, supported by enhanced assimilate translocation, conferred HNT tolerance in rice. Thus, dynamic proteome programming across key developmental stages guides future crop improvement efforts. The metabolomic analysis provided potential markers for breeding stress-tolerant rice germplasm (Lawas et al., 2019).

## 8 How does the rice plant respond to HTS at the molecular level?

**Figure 6:**
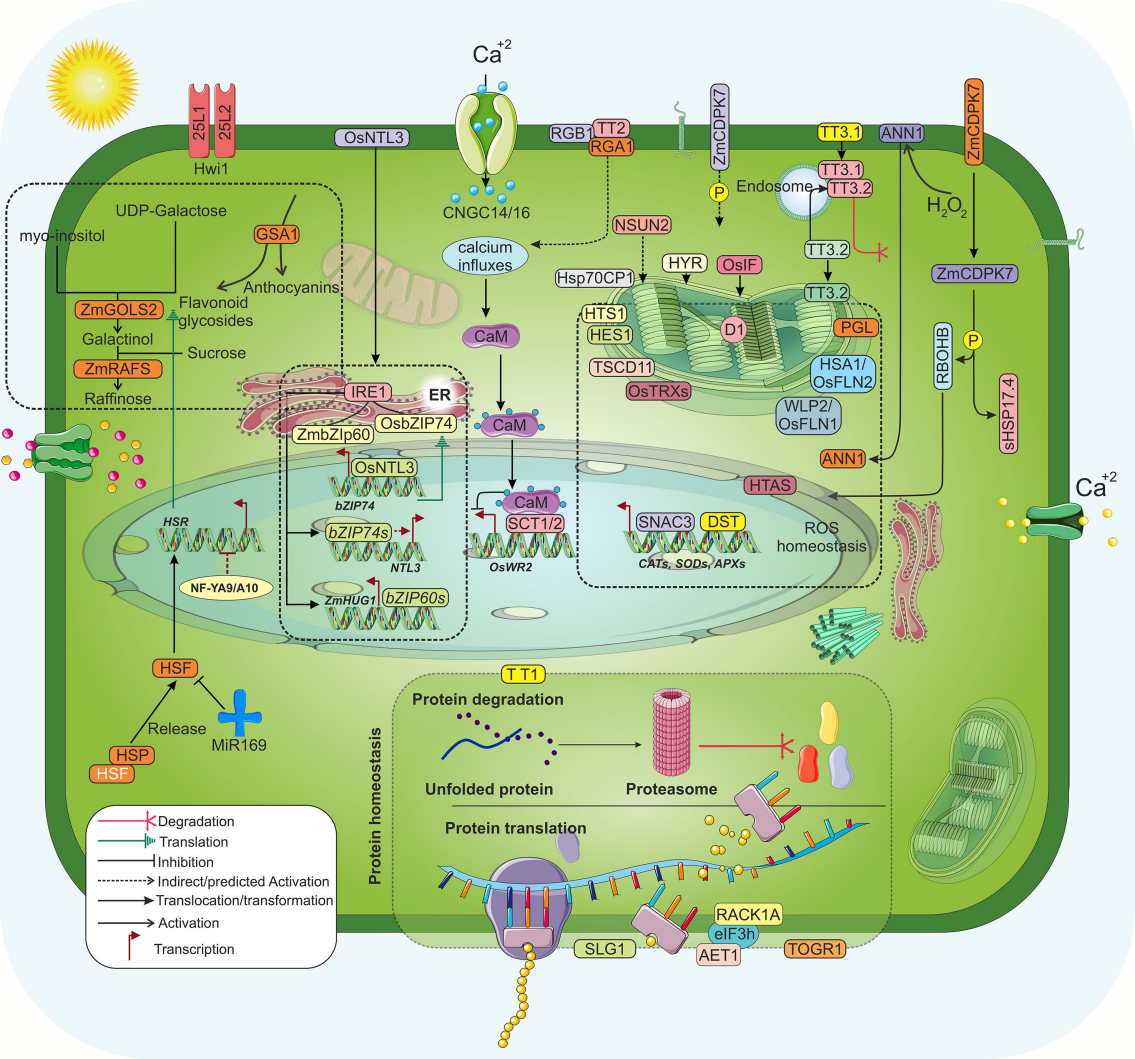
General molecular responses to HS in rice and other crop plants. The cell wall responds first to HS and plays a crucial role in thermal sensing. Ca^2+^ signal induction is the most rapid response. The calcium signaling pathway is crucial for activating, inhibiting, and regulating various Ca²^+^/calmodulin-dependent kinases (CaMKs), phosphatases, and numerous TFs. Hwi1 acts as potential receptor-like kinase that function upstream in the heat-response pathway. Cyclic nucleotide-gated channels OsCNGC14 and OsCNGC16 respond to HS by triggering Ca^2+^ signals. *TT2* facilitates the heat-induced rise in cytosolic Ca^2+^ levels, which is then interpreted through Cam-SCT1/2 interactions, suppressing *OsWR2* (*Wax Synthesis Regulatory2*) transcription. *RGB1* (encodes a β-subunit of G protein) reduces electrolyte leakage and the accumulation of MDA and H_2_O_2_. *RGA1* encodes the Gα subunit of the G protein and is involved in signal transduction. During HS, TT3.1 moves from the plasma membrane to the endosomes, where it recruits TT3.2 and facilitates its degradation through ubiquitination, thereby preventing the accumulation of mature TT3.2 in the chloroplast and protecting thylakoids. In response to HS, ZmCDPK7 (calcium-dependent protein kinase) translocates from the PM to the cytosol and phosphorylates sHSP17.4 (small HSP 17.4) and RBOH (a key producer of ROS), enhancing their expression levels. HS triggers translocation of OsNTL3 (a NAM, ATAF1/2, CUC1/2[NAC] transcription factor) from PM to the nucleus and activates transcription of downstream genes. *ANN1* (annexin1), SNAC3, DST, and HTAS are pivotal in the scavenging of ROS by regulating the accumulation of key antioxidant enzymes like CATs (Catalases) and SOD (Superoxide dismutases) and APXs (Ascorbate peroxidases). The endoplasmic reticulum-localized TFs OsbZIP74 and ZmbZIP60 are spliced by IRE1 and are involved in the transcriptional activation of *OsNTL3* and *ZmHUG1(Heat Up-Regulated Gene 1)*. The PSII components D1, *OsIF* (encodes intermediate filament protein), *PGL* (encodes chlorophyllide a oxygenase 1), and HES1 (UGPase enzymatic activity) are essential for preserving chloroplast ultrastructure. During HS conditions, TSCD11 (seryl t-RNA synthetase) and Hsp70CP1 (chloroplast localized Hsp70) regulate the chloroplast development. HYR, a transcription factor and NSUN2 (RNA methyltransferase) ensure the transcripts of the photosynthetic gene, while HTS1(β-ketoacyl carrier protein reductase localized in thylakoid membrane) regulates lipid metabolic flux. OsTRXz–OsFLN2–OsFLN1 complex (encoding thioredoxin protein and fructokinase-like proteins), PGL, HTS1, HES1, and TSCD11 work together to maintain redox balance in the chloroplast. TT1 and HTAS remove unfolded proteins, while the RACK1A–eIF3h–AET1 (Receptor for Activated C Kinase 1A- eukaryotic Initiation Factor Subunit h- Adaptation to Environmental Temperature 1) complex, SLG1, and TOGR1 ensure protein translation under HS. In response to HS, the HSP-HSF complex dissociates, releasing HSFs. This release removes the NF-YA9/A10 (Nuclear Factor Y subunit A9/A10) - mediated inhibition of heat shock response (HSR) through HSF-driven accumulation of miR169s. GSA1 enhances thermotolerance in rice by regulating the flux of flavonoid glycosides and anthocyanins. In maize, GOLS2 and RAFS are transcriptionally activated by HSF, which enhances raffinose biosynthesis to modulate heat tolerance.

A slight increase in average temperature may not cause severe damage to cells, tissues, and heat shock responses (HSR). Still, it may cause morphological changes, biorhythms, and immunity response (Shrestha et al., 2022). The cell wall is the first protective barrier in plants and responds first to HS, followed by changes in membrane fluidity and activation of channel receptors, which play an essential role in thermal sensing, cellular response, and calcium signal transduction. Increased ROS levels prompt the release of Ca^2+^ from different organelles, resulting in the sudden release of apoplastic Ca^2+^ and raising the amount of free Ca^2+^ in the cytoplasm, which performs an essential role in activating or repressing Ca^2+^/Cam-related kinases, phosphatases, and transcription factors (TFs) (Kan and Lin, 2021). The cell wall remodeling protein, pectin methylesterase (PME), can have its expression upregulated by HS, which increases the amount of demethylesterified pectin. PME activity and pectin demethylesterification are escalated by increased H_2_O_2_ content, which ultimately activates the downstream regulatory pathway of HS response (Wu et al., 2018; Xu et al., 2021). *OsCNGC14* and *OsCNGC16* responded to HS by triggering calcium signals, and both mutants (*oscngc14* and *oscngc16*) exhibited impaired Ca^2+^ influx to the cytosol and decreased or abolished cytosolic Ca^2+^-mediated signal transduction in response to HS (Cui et al., 2020) (**Figure 6**). Although *OsCNGC9* and *OsCNGC13* channels are likewise Ca^2+^ permeable and involved in cytosolic Ca^2+^ mediated signaling cascades, additional research is essential to fully elucidate their roles in HS response (Wang et al., 2019; Xu et al., 2017). The *OsHsfA2e* gene has been observed to be associated with a CaMK engaged in the Ca2+/calmodulin-dependent signaling pathway, and its interaction with *Hsp70* and *Hsp90* is crucial in the response to HS (Harshada et al., 2021). Changes in membrane fluidity induced by heat may activate PM-bound phospholipases and kinases, leading to rapid increases in phosphatidic acid and phosphatidylinositol diphosphate (PIP2), which are vital in stress signal transduction (Niu and Xiang, 2018).

During HS, ER-localized *OsbZIP74* translocates to the nucleus, initiating the expression of *OsNTL3*, followed by the transport of membrane-localized *OsNTL3* to the nucleus to regulate the expression of *OsZIP74. OsNTL3* encodes a NAC TF with a predicted C-terminal transmembrane domain (Liu et al., 2020; Lu et al., 2012). The repair of damaged PSII and the PGL gene (pale green leaf, encoding chlorophyllide and oxygenase 1) in the chloroplast requires the *de novo* synthesis of the D1 subunit (Kan and Lin, 2021); meanwhile, the OsTRXz-OsFLN1/2, encoding thioredoxin protein and fructokinase-like proteins, respectively, complex shields chloroplasts from heat-induced damage (Lv et al., 2017) (**Figure 6**) and the mitochondria-localized EG1 protects the organelle and maintains floral robustness (Zhang et al., 2016).

The most significant elements of the intricate transcriptional regulatory network of HSR in plants are heat shock TFs, or heat shock transcription factors (HSFs). They initiate a transcriptional cascade that activates genes encoding HSR-induced TFs, ROS-scavenging enzymes, metabolic enzymes, and HSPs (Gong et al., 2020). Among 25 HSFs in rice, HT stimulates the expression of 22 of these genes (Mittal et al., 2009; Xu et al., 2021). Among these HSFs, HSFA1s are considered ‘‘master regulators” in the transcriptional network (Ohama et al., 2017). When rice plants experience HS, alternative splicing induces the transcriptionally active form of *OsHSFA2d*, which increases the expression of genes like *HSP17.7, HSP18.2, HSP21, HSP83.1, and HSP101* (Cheng et al., 2015). The isolated proteins OsHSP17.4 and OsHSP17.9A exhibit chaperone activity by effectively preventing the aggregation of proteins (Sarkar et al., 2019).

## 9 Genetics of rice thermotolerance: key QTLs and candidate genes

HS tolerance is often viewed as a quantitative trait, and despite considerable research over the past several years, the genetic basis of HS tolerance remains largely unclear. In recent years, advancements in molecular marker technology and increased research focus on rice heat tolerance have identified numerous heat-tolerant QTLs across 12 rice chromosomes. Identifying QTLs for rice thermotolerance has been hampered by several issues, such as inconsistent phenotyping, lack of genetic resources with established thermotolerance, trait complexity, and environmental factors. Notwithstanding these obstacles, rice QTL mapping for heat tolerance has advanced significantly.

### 9.1 QTLs identified for thermotolerance in rice

The detection of QTLs helped understand rice’s genetic mechanism, marker-assisted selection, and QTL cloning (Khan et al., 2019). Efforts for molecular mapping of rice thermotolerant QTLs have been carried out at booting, flowering, and grain filling to ripening stages (Buu et al., 2021; Liu et al., 2023). Most recently identified heat-tolerant QTLs pertain to these stages, with fewer associated with seedling stages (**Table 2**). A natural QTL, *TT2* (*THERMOTOLERANCE 2*), encoding a Gγ subunit, reduces yield losses under HS at both vegetative and reproductive stages. It regulates G protein, wax metabolism, and Ca^2+^ signaling, potentially reducing the yield penalty under HS (Kan et al., 2022) (**Table 2**). Major QTL, *TT1-2*, was controlled by a single dominant gene. It was narrowed to a 26.0 kb region (Yan et al., 2021). The QTL *qEMF3*, identified from wild rice *O. officinalis*, holds a very promising role in shifting rice cultivars’ flower opening time to earlier in the morning (Hirabayashi et al., 2015). By creating a mapping population using a common wild rice as one of the parents, heat-tolerant QTLs *qHTH5, qHTH10*, and *qHTB1–1* were detected. At the heading and flowering stage, QTL *qHTH5* was located within 304.2 kb on chromosome 5 short arm (Hu et al., 2022). SNP markers were utilized for mapping two major QTLs, namely *qHTSF1.1* and *qHTSF4.1*, on chromosomes 1 and 4, respectively by generating the population from a cross IR64/N22 for spikelet fertility variation (Ye et al., 2011). Two major QTLs for chalkiness, *qPGC5* and *qPGC6*, were located by substitution mapping of single-segment substitution lines (SSSLs). *qPGC5* was located in the 876.5 kb interval of chromosome 5, and *qPGC6* was located in the 269.1 kb interval of chromosome 6 (Yang et al., 2022). Fengfeng et al. (2023) mapped three heat tolerant QTLs at seedling stage from *O. longistaminata*, including novel heat tolerant loci *qTT4* and *qTT5*. Two QTLs for spikelet sterility with significant genetic effects, *qSTIPSS9.1* and *qSTIY5.1/qSSIY5.2*, were located within genomic regions below 400 kbp (PS et al., 2017). Park et al. (2020) utilized the double-haploid line derived from Cheongcheong/Nagdong to investigate the QTL associated with booting stage thermotolerance (**Table 2**). Major QTLs *qHAC4, qHAC8a*, *qHAC8b*, and *qHAC10* minimize the adverse impact of HT on amylose content by upregulating the splicing efficiency of the *Wx* gene (Zhang et al., 2014). Eleven QTLs associated with thermotolerance at anthesis were detected by Zhao et al. (2016), further confirming *qPSL^ht^4.1* across various temperature conditions. This locus has been consistently detected in multiple studies. Numerous QTLs contributing to heat tolerance at grain filling stage have also been mapped. For instance, the *Appearance quality of brown rice 1 (Apq1)* QTL has been localized to a 19.4-kb region, with the underlying gene identified as *sucrose synthase 3 (Sus3)* (Takehara et al., 2018).

**Table 2:** Major QTLs governing stress tolerance in rice.

QTL	Chromosome number	Trait	Mapping population	Marker type	Donor	Reference
*qADL09-5/qADL10-5*	5	Apical dehiscence length	BILs	RFLP	Kasalath	Tazib et al. (2015)
*qEMF3*	3	Flower opening time	F_2_	SSR	EMF20	Hirabayashi et al. (2015)
*qHTSF4.1*	4	Spikelet sterility	BC_5_F_2_	SNP	N22	Ye et al. (2015)
*qTT1*	3	Elimination of cytotoxic denatured proteins	BC_4_F_2_	SNP	CG14 (*O. glaberrima*)	Li et al. (2015)
*qBDL11, qBDL2-1*	11, 2	Basal dehiscence length	BC_4_F_2_	SSR	Nipponbare	(Zhao et al., 2016)
*qSSIY5*.2	9	Stress susceptibility index	F_7:8_	SNP	N22	PS et al. (2017)
*qSTIPSS9.1*	9	Spikelet sterility	F_7:8_	SNP	N22	PS et al. (2017)
*qHT-3, qHT-6, qHT-8, qHT-12*	3,6,8,12	Spikelet fertility	SSSLs		Gan-Xian-Nuo	Liu et al. (2017)
*qMW4.1*	4	Milky white grains	BC_4_F_2_	SSR	Chikushi52	Miyahara et al. (2017)
*qHTB3-3*	3	Spikelet fertility	Chromosome segments substitution lines (CSSL)	SSR	Habataki	Zhu et al. (2017)
*qDHT 1, qDHT 5, qDHT 7*	1, 5,7	Seed germination	F_18_	RFLP, SSLP, AFLP	Milyang 23	Lee et al. (2017)
*rlht5.1*	5	Root length	F_8_	SNP	N22	Kilasi et al. (2018)
*qNS1, qNS4, qNS5, qNS6*	1,4,5,6	Seed setting	F_8_	SSR	Liaoyan241	Li et al. (2018)
*qUSN10.2*	10	Unfilled spikelet number	BC_1_F_8_	SNP	Koshihikari	Seo et al. (2019)
*qSF1, qSF2, qSF3*	1,2,3	Spikelet fertility	F_2_	SNP	M9962	Nubankoh et al. (2020)
qHdd1 and qHdd1-2	1	Heading date	Double haploid	SSR	Nagdong	Park et al. (2020)
qCl11 and qCl11-2	11	Culm length				
qPl1, qPl1-2, and qPl12	1,12	Panicle length				
qNt1	1	Tiller number				
qTgw1, qTgw1-1, and qTgw12	1, 12	1000 grain weight				
qCc1 and qCc1-3	1	Chlorophyll content				
*qHTB1*-*1*	1	Spikelet fertility	BC_5_F_2_	SSR	Hehuatang No. 4 (*Oryza rufipogon*)	Cao et al. (2020)
*qHHT8*	8	Spikelet fertility	F_2:3_	SNP	Huanghuazhan	Chen et al. (2021)
*qFW6, qFW11.1, qFW11.2*	6,11,11	Fresh weight of seedling	RIL	SNP	PA64s	Wei et al. (2021)
*qSL12.1, qSL12.2*	12	Shoot length				
*qDW11, qCDW11*	11	Dry weight of seedling				
*qSSR7-1, qSSR11-1*	7, 11	Seed setting rate	BC_4_F_4_	SSR	N22	Nguyen et al. (2022)
*THERMOTOLERANCE 2 (TT2)*	3	Encoding Gγ subunit (Wax biosynthesis)	BC_5_F_2_	SNP	HP21 *(O*. *glaberrima)*	Kan et al. (2022)
*TT3*	3	Transduces heat signals from PM to chloroplasts	NILs	SNP	CG14	Zhang et al. (2022)
*qTT4*, *qTT5*, *qTT6*	2, 8, 9	Seedling survival rate	BC_2_F_20_	SNP	IRGC103886 *(O. longistaminata)*	Fengfeng et al. (2023)
*qHD8*	8	Heading date	F_7_	SSR, STS	Zhonghui 161	Huang et al. (2023)
*qSF1*, *qSF2*, *qSF3.1*, *qSF3.2*, *qSF8*	1, 2, 3,3, 8	Spikelet fertility				
*qGL-HNT-1, q%Chalk-HNT-6, q%Chalk-HNT-7*	1, 6, 7	Grain quality	F_12_	SNP	Cypress	Kumar et al. (2023)

### 9.2 Candidate genes and molecular insights for heat tolerance

Two major candidate genes *LOC_Os08g07010* and *LOC_Os08g07440* identified within a major QTL *qHTT8*, on chromosome 8, controlling flowering stage thermotolerance (Chen et al., 2021). Gene *LOC_Os09g38500* within the novel QTL *qRSF9.2* region is associated with controlling relative spikelet fertility under HS (Hu et al., 2022). The *qHTB1–1* QTL, controlling thermotolerance at the booting stage in rice, was fine-mapped to a 47.1 kb region containing eight candidate genes. Two positional candidate genes (*LOC_Os01g53160* and *LOC_Os01g53220)* showed significant changes in expression levels under HS (Cao et al., 2020). *OsHTAS* codes for a ubiquitin ligase found in both the nucleus and cytoplasm. It reacts to various stresses and shows robust activation in response to externally applied ABA. *OsHTAS* regulates the accumulation of H_2_O_2_ in shoots, affects the opening of stomata on rice leaves, and enhances ABA biosynthesis (Liu et al., 2016) (**Table 3**). Candidate genes *LOC_Os04g52830* and *LOC_Os04g52870* located within the locus *qHTT4.2* enhance seed setting rate under HS (Pan et al., 2023). Das et al. (2024) carried out genome-wide association mapping and found three significant QTLs and three promising putative candidate genes regulating the Photosystem II (PSII) complex impairment during HS. Common wild rice (*O. rufipogon* Griff.) is a valuable source of germplasm that can be used to improve rice. The genetic population created with the common wild rice (*O. rufipogon* Griff.) as a parent is a robust QTL mapping population for HS tolerance. In addition, common wild rice is a significant source of high-temperature tolerant rice germplasm (Ishimaru et al., 2010; Xiao et al., 1996).

**Table 3:** Stage specific genes involved in HS tolerance across growth stages in rice.

Gene	Chromosome number	Expression	Function	Expression tissue	Stage	Reference
*OsNox5, OsNox6, OsNox7, OsNox8, OsNox9*	5, 6, 9, 11, 12	Upregulated	ROS-dependent plant immune response	Roots, shoots, leaf blades, leaf sheath, panicles	Tillering and heading	Wang et al. (2013)
*OsNox1, OsNox2, OsNox3*	1	Downregulated				
*OsFRO1*	4					
*Apq1 (Appearance quality of brown rice 1)*	7	Upregulated	Improves the quality of ripening grains	Grain	Ripening	Murata et al. (2014)
*OsHSFA2d*	3	Upregulated	Transcriptional activator, involved in unfolded protein response signaling pathway	Leaves	Seedling	Cheng et al. (2015)
*OsTT1*	3	Upregulated	Elimination of cytotoxic denatured proteins	Extensively	Seedling	Li et al. (2015)
*SNAC3*	1	Upregulated	Modulate ROS homeostasis.	Leaves	Seedling	Fang et al. (2015)
*OsANN1*	2	Upregulated	Modulating the production of hydrogen peroxide and maintaining redox homeostasis	Extensively	Seedling, Reproductive	Qiao et al. (2015)
*OsHTAS (Oryza sativa-HEAT TOLERANCE AT SEEDLING STAGE)*	9	Upregulated	Modulation of H_2_O_2_-induced stomatal closure	All tissues	Seedling	Liu et al. (2016)
*TCM5*	5	Upregulated	Encodes chloroplast-targeted Deg protease protein, which is important for chloroplast development and the maintenance of PSII function	All green tissues	Seedling	Zheng et al. (2016)
*EG1 (EXTRA GLUME1)*	1	Upregulated	Encodes a predominantly mitochondrial functional lipase, acts upstream of several floral identity genes, and promotes floral robustness	Spikelets	Flowering	Zhang et al. (2016)
*OsHsp18.0*	3	Upregulated	Small heat-shock protein, nucleo-cytoplasmic trafficking	Shoot and root	Seedling	Kuang et al. (2017)
*HSA1*	3	Downregulated	Encodes fructokinase-like protein 2 (FLN2), Regulates and protects chloroplast development under HS	Chloroplast	All stages	Qiu et al. (2018)
*LS1*	11	Upregulated	Protect genome stability and leaf structure from high light and HT	Extensively expressed	Seedling	Qiu et al. (2019)
*OsPL*	5	Upregulated	Negatively control anthocyanin accumulation and alteration of hormone signaling	Seeds, roots	Late grain filling stage	Akhter et al. (2019)
*AET1*	5	Upregulated	Associated with translation regulation, modification of pre-tRNA^His^, regulates auxin signaling in response to HS	Shoot and root	Tillering	Chen et al. (2019)
*OsSPL7*	5	Upregulated	Maintain ROS balance via the regulation of downstream gene expression	Leaves	Seedling	Hoang et al. (2019)
*OsCNGC14, OsCNGC16*	3, 5	Upregulated	Cytosolic Ca^2+^ -mediated signaling	PM	Seedling	Cui et al. (2020)
*cpHSP70-2*	12	Upregulated	Chloroplast heat-shock protein, lower the level of chalkiness under HS	Seeds	Grain ripening	Tabassum et al. (2020)
*OsBIP2*	3	Upregulated	Thermotolerance of pollen tubes	Reproductive tissues	Flowering	Raza et al. (2020)
*OsNTL3*	1	Upregulated	Encodes a NAC TF, regulates expression of genes associated with ER protein folding	Leaves, Roots	Seedling	Liu et al. (2020)
*OsBHT (Oryza sativa booting stage high-temperature tolerance)*	1	Upregulated	An Hsp-p23-like calcyclin-binding protein that is a type of HSP	Extensively	Booting	Park et al. (2020)
*OsNSUN2*	9	Upregulated	RNA 5-methylcytosine (m^5^C) methyltransferase, involved in protein degradation and RNA and chloroplast homeostasis	Root, shoot, and leaves	Seedling	Tang et al. (2020)
*SLG1 (Slender Guy 1)*	12	Upregulated	Encodes cytosolic tRNA 2-thiolation protein 2 (RCTU2)	Extensively	Seedling and reproductive	Xu et al. (2020)
*OsACT*	3	Upregulated	Stable anther structure under HT	Anthers	Anthesis	Liu et al. (2020)
*OsHSP26.7*	3	Upregulated	Heat-shock protein	Leaves and flowers	Flowering	Harshada et al. (2021)
*OsSFq3*	3	Upregulated	Formation and breakdown of amylose and amylopectin influence spikelet fertility and overall grain quality	Pollen, Grains	Flowering, Grain filling	Park et al. (2021)
*OsABI2, OsPP2C09, OsPP2C68, OsbZIP18*	1, 1,9, 2	Downregulated	Regulate the transcription of genes encoding negative regulators of ABA signaling	Universally expressed	Seedling, booting	Sharma et al. (2021)
*OsPP2C51*	5,	Upregulated	ABA signal transduction			
*OsIAA20*	6	Upregulated	Aux/IAA encoding gene, leading to downregulation of ARFs			
*OsDi19-1*	5	Upregulated	Involved in auxin-mediated response			
*ILL8 (ILR-like 8)*	7	Upregulated	Codes for IAA-leucine hydrolase			
*eIF4A1*	6	Upregulated	RNA splicing, ribosome biogenesis, and RNA degradation	Root and shoot	Seedling	Singha et al. (2021)
*OsC3H60*	9	Upregulated	Encodes a U2 snRNP auxiliary factor small (35 kDa) subunit A, which undergoes significant heat-induced alternative splicing	Leaves, roots, and stem	Seedling	Vitoriano and Calixto (2021)
*ONAC127, ONAC129*	11	Upregulated	Regulate stimulus-response and nutrient/sugar transport.	Seeds	Grain filling	Ren et al. (2021)
*LOC_Os01g09450, LOC_Os03g59040*	1, 3	Downregulated	Auxin signaling pathway, squalene synthase involved in the biosynthesis of sterols impacting structural and functional integrity of membranes	Leaves and roots	Seedling	Wei et al. (2021)
*OsSAP5*	2	Upregulated	Spermidine (Spd)-mediated enhancement of thermotolerance and seed quality	Seeds	Seed development	Chen et al. (2021)
*TT3.1, TT3.2*	3	Upregulated	Transduces heat signals from PM to chloroplasts and protects thylakoids from HS	Extensively	Seedling	Zhang et al. (2022)
*OsDREB1C/E/G*	6, 4, 2	Upregulated	Essential for signal transduction and expression activation of stress responsive genes	Extensively	Seedling	Wang et al. (2022)
*OsDjA7*,	5	Upregulated	DNA replication and repair, chloroplast development	Leaves	Seedling	Wang et al. (2022)
*OsGrp94*	6		Form a caspase–3–related protein complex in rice suspension cells, increase innate immunity			
*OsGSK1*	1		Brassinosteroid (BR) signal transduction			
*OsALDH5F1, OsALDH7*	2, 9		Acetaldehyde dehydrogenase oxidizes toxic aldehydes into corresponding non–toxic carboxylic acids, maintains the balance of aldehydes			
*OsBADH1*	4		Betaine aldehyde dehydrogenase gene, modulate oxidation of acetaldehyde produced by catalase			
*LOC_Os01g04580*	1	Downregulated	A Ser/Thr protein kinase, putative gene	Extensively	Flowering and maturity	Ravikiran et al. (2022)
*HTH5*	5	Upregulated	Encode pyridoxal phosphate homeostasis protein, decreases ROS level by elevating heat-induced pyridoxal 5’-phosphate (PLP)	Mitochondria	Heading stage	Cao et al. (2022)
*HES1 (High-Temperature Enhanced Lesion Spots 1)*	8	Upregulated	Encodes a UDP-N-acetylglucosamine pyrophosphorylase, reduces DNA and chloroplast damage	Leaves	Seedling, booting, and maturing	Xia et al. (2022)
*Os01g0180800*	1	Downregulated	Encode storage proteins, late embryogenesis abundant proteins, and unspecified proteins	Panicles, seed	Maturity	Zhou et al. (2022)
*Os11g0703900*	11	Downregulated				
*Os12g0244100, Os12g0569700*	12	Downregulated				
*LOC_Os07g48710*	7	Upregulated	Encoding a VQ domain-containing protein	Shoot	Seedling	Li et al. (2023)
*SRL10*	10	Upregulated	Encodes a dsRNA binding protein, regulates leaf morphology and thermotolerance through alteration of microRNA biogenesis	Leaves	Seedling	Wang et al. (2023)
*CNX, CRT, Ero1, BiP, GRP94*	4, 1, 3, 8, 6,	Upregulated	Involved in the misfolded protein repair	Leaves	Seedlings	He et al. (2023)
*OsGRF4 (Oryza sativa growth-regulating factor 4)*	2	Upregulated	Negatively affects the proper transcriptional and splicing regulation of genes under HS	Pollen and seeds	Flowering and seed setting	Mo et al. (2023)
*TTL1 (Thermo-Tolerance and grain Length)*	1	Upregulated	Negative regulator of heat tolerance with pleiotropic effects, functions as a transcriptional regulator	Expressed widely	Seedling and grain formation	Lin et al. (2023)
*LOC_Os06g23160*	6	Upregulated	Encodes a bacterial transferase hexapeptide domain containing protein.	Extensively	Seedling	Xie et al. (2024)
*OsIAA29*	11	Upregulated	Mediates auxin signaling pathway, regulates expression of several starch and protein synthesis-related genes	Seeds	Grain filling	Chen et al. (2024)

Knockout mutants of *OsRboh* (NADPH oxidase) showed upregulation of heat responsive genes in seedlings of Nipponbare (Liu et al., 2025). At seedling stage *OsIAA7* increases thermotolerance by positively affecting malondialdehyde, catalase, and chlorophyll A levels. It decreases H_2_O_2_ levels and prevents cell death. *OsARF6 (auxin response factor gene)* negatively modulates heat tolerance by influencing the expression levels of *OsTT1* and *OsTT3.1* (Qiu et al., 2025). Two locus namely *qDW7* (dry weight) and *qFW6* (fresh weight) showed association with rice response to HT. Localized genes such as LOC_Os06g10790, LOC_Os07g30330, and LOC_Os03g59040 were found responsive to HS at seedling stage (Wei et al., 2021). Expression level of genes (*ethylene insensitive 2, ethylene insensitive-like1*, and *ethylene insensitive-like 2*) involved in ethylene signaling, is increased under HS in rice seedlings (Wu and Yang, 2019).

In seeds developed during HS, the promoter regions of starch biosynthesis genes *OsAGPS2b* (*ADP-GLc pyrophosphorylase subunit 2b*), *OsGBSSI* (*granule-bound starch synthase*), and *OsSuSy2* (*sucrose synthase 2*) showed marked hypomethylation. In contrast the promoters of α-amylase genes *OsAmy1A* and *OsAmy3D* were significantly hypermethylated (Suriyasak et al., 2025). A promotor variant of WCR1 (White-Core Rate 1) increases OsDOF17 binding, leading to higher WCR1 expression, which in turn reduces chalkiness and enhances grain quality (Wu et al., 2022). HS upregulated the expression of genes associated with NADPH oxidases *(OsRbohB, OsRbohD, OsRbohF*, and *OsRbohl*) and GA biosynthesis (*OsGA30x1 and OsGA20ox1*) during grain filling. The *GSA1* (*Grain Size and Abiotic Stress tolerance 1*) locus, which encodes a UDP-glucosyltransferase, controls grain size by influencing cell proliferation and cell expansion (Dong et al., 2020). Genes *ONAC127 and ONAC129*, encoding TFs are expressed in pericarp modulating sugar transport during HTS at grain development stage (Ren et al., 2021) (**Table 3**).

## 10 Heat stress-induced epigenetic modification in rice

In addition to morphological and molecular adaptations, plants also adapt through epigenetic mechanisms, which involve DNA methylation, chromatin modeling, histone modification, sRNAs, and lncRNAs for surviving under adverse environmental conditions by altering the gene expression pattern and/or epigenetic memory (Crisp et al., 2016; Kinoshita and Seki, 2014; McCormick, 2018). Epigenetics has a potential role in increasing thermotolerance, as demonstrated by the growing acceptance of its application in HS resilience breeding (Gardiner et al., 2015; Yadav et al., 2022).

During rice seed development, moderate HS causes the demethylation of the *OsFIE1* (*O. sativa Fertilization-Independent Endosperm1)* locus and represses *CMT3* (*CHROMOMETHYLASE3*) expression, leading to correlated increase in *OsFIE1* transcript levels. This increase is associated with a decrease in repressive H3K9me2 mark on *OsFIE1.* Enhanced *OsFIE1* activity (as part of Polycomb Repressive Complex 2) then deposits the repressive H3K27me3 (Histone H3 lysine 27 trimethylation, an epigenetic modification) mark on target genes like *MADS82* and *MADS87*, (MADS box TFs), which ultimately causes precocious cellularization and reduced seed size (Folsom et al., 2014). The indica rice variety 93–11 shows more dynamic changes in chromatin accessibility and gene expression under HS than the japonica variety Nipponbare, aligning with the greater heat tolerance observed in 93–11 relative to Nipponbare (Liang et al., 2021). Genome-wide survey of histone H3 lysine4 tri-methylation (H3K4me3) under drought conditions revealed differential methylation of 4837 genes, out of which 3927 showed increased expression while 910 showed decreased total transcript. HS during grain filling significantly increased DNA methylation of promotors in ABA metabolism related genes (*OsNCED2, OsNCED3*, and *OsNCED5*), ABA catabolism genes *(OsABA8′OH1*, *OsABA8′OH2*, and *OsABA8′OH3*), and α-amylase genes (*OsAmy1C, OsAmy3B*, and *OsAmy3*) causing delayed germination of heat stressed seeds. Predicted CpG islands were found in these genes except in genes *OsNCED2, OsABA8′OH2*, or *OsAmy1A.* Presence of CpG islands and hyper-methylation in α-amylase promotors cause HS induced transcriptional regulation during seed imbibition (Suriyasak et al., 2020). Under HS, rice roots and shoots displayed varied expression levels of miR160 and miR169, indicating that heat regulates target genes differently in these two distinct tissues (Sailaja et al., 2014). Elevated temperatures during early seed development prompted genome-wide alterations, including the reactivation of transposable elements by a decrease in DNA methylation in noncoding regions (Chen et al., 2016). Rice lines overexpressing *tae-miR159* and *Arabidopsis myb33myb65* double mutants exhibit increased sensitivity to HS compared to their wild-type counterparts. This suggests that the down-regulation of *miR159* and the subsequent up-regulation of its target genes following HS may be involved in a HS-related signaling pathway, contributing to HS tolerance (Wang et al., 2012). Li et al. (2023) applied methylation-sensitive amplification polymorphism31 (MSAP) to investigate the DNA methylation responses in rice spikelets at the anthesis stage under control and HT conditions. DNA methylation level significantly increased in the susceptible rice group and decreased in the tolerant rice group under HT treatment. Genes in N22 leaves with hypomethylation that exhibit up-regulated expression under direct seeded rice circumstances, implying that epigenetic alteration plays a significant role in N22’s adaptive plasticity. The ability of N22 to adapt to unfavorable climatic conditions is attributed to variations in chromatin architecture and post-translational modification of proteins, including histone modifications (Seem et al., 2024). Liu et al. (2017) mapped four QTLs (*qHT-3, qHT-6, qHT-8*, and *qHT-12*) for thermotolerance during flowering. Their detailed analysis revealed that these QTLs contained miRNA targets associated with ABA-responsive genes. Moreover, they discovered that the suppressor of the G2 allele of *skp1 (SGT1)*, a direct target of *miRNA166e*, was located within the *qHT-8* locus. Studies of the mechanism and nature of these various epigenetic modifications can provide valuable insights into the different genes and the specific regions in them responsible for adapting to HTS, leading to the development of a better understanding of the pathway to be targeted for thermotolerance breeding.

## 11 Breeding to improve thermotolerance in rice

Among the various strategies aimed at alleviating the impact of HS on rice, breeding emerges as a fundamental approach. Development of heat-tolerant rice cultivars through thermotolerance targeted breeding programs presents a cost effective, long-lasting and sustainable solution. Relative to other abiotic stresses like drought and salinity, breeding endeavors for heat-tolerant rice varieties have attracted less research and focus. There is inadequate data concerning HS resistant rice cultivars and the genetic mechanisms underlying their tolerance. Moreover, the identified genetic resources and QTLs have not been comprehensively employed in breeding programs to develop thermotolerant rice varieties (Stephen et al., 2022).

### 11.1 Conventional breeding

Conventional breeding typically relies upon extensive phenotypic characterization, selecting phenotypes related to thermotolerance, and is conducted in regions with climates similar to where the crop will be cultivated (Driedonks et al., 2016). Assessing thermotolerance levels precisely, selecting superior breeding lines, and effectively transferring traits related to heat tolerance into specific cultivars with favorable agronomic traits are crucial aspects of conventional breeding. The straightforward traits, such as pollen fertility, seed setting rate, spikelet fertility, grain chalkiness, etc., serve as reliable indicators in conventional breeding for heat tolerance. Using these indicators, a large number of heat-tolerant rice genotypes have been identified and some has been utilized as donors to develop breeding lines, including N22 (Kilasi et al., 2018; Ye et al., 2015), Giza178 (Abdelaal et al., 2021), IR2061 (Cheng et al., 2012), and (Zhao et al., 2016) (**Table 2**). Some heat-tolerant hybrid rice varieties, such as Guodao 6, maintain seed-setting stability under HS conditions due to their adaptability to heat avoidance, characterized by floral traits like shortened flowering phases (Tao et al., 2008). N22 is frequently employed as a control in studies focusing on heat tolerance. Giza178, originating from a *japonica-indica* cross and hailing from Egypt, demonstrates notable heat tolerance (Tenorio et al., 2013). Interspecific rice hybrid NERICA-L-44 has been characterized as a heat-tolerant variety (Bahuguna et al., 2015). Using ^20^Gy proton, three heat-tolerant mutant lines 8852, 8552, and LP-12 were developed utilizing varieties J-104 and A-82 through a mutation breeding approach (Gonzalez et al., 2021).

### 11.2 Marker-assisted selection for thermotolerance in rice

**Figure 7:**
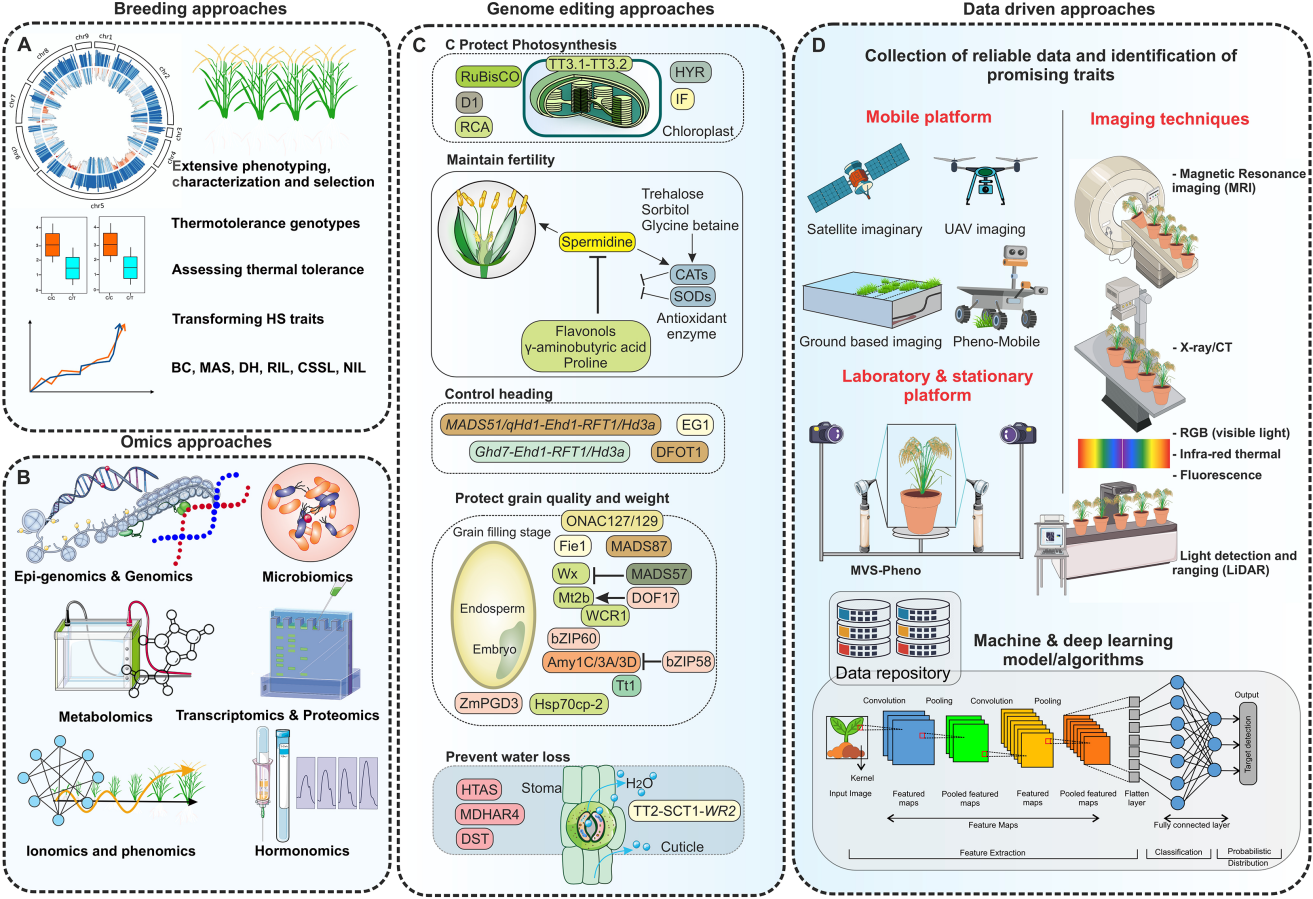
Strategies to develop thermotolerance in rice. **(A)** Breeding approaches: Backcross (BC), marker-assisted selection (MAS), double haploid (DH), recombinant inbred lines (RILs), near-isogenic lines (NILs), and chromosome segments substitution lines (CSSLs) breeding methods are used extensively to develop varieties with desirable traits, including thermotolerance. Extensive phenotyping, characterization, and selection of germplasm for heat tolerance, assessing the thermotolerance level, and transferring HS tolerance into agronomically superior cultivars are the significant components of breeding for heat tolerance. **(B)** Omics Approaches: The incorporation of different omics technologies could broaden our understanding of molecular and biochemical insights into the complex interplay among genes, pre- and post-translational modifications, including DNA methylation, expressed proteins, and metabolites. These technologies help in the identification of heat-responsive QTLs, genes, proteins (HSPs, chaperones, dehydrins, LEA, etc.), and metabolites (prolines, sugars, spermidine, betaine, phenolic compounds, amino acids, and lipid-derived metabolites such as jasmonic acid). Hormonomics is a key tool for deep physiological phenotyping and could provide insights into cellular signaling, facilitate gene discovery, and allele mining. It is crucial to understand the microbiome and its interaction with the host under HS conditions. Deciphering whole genomes of microorganisms, i.e., microbiomics, is essential to uncovering the complex interaction between plants and microbes under stress conditions. **(C)** Genome editing (GE) approaches: GE is one of the most promising approaches to understanding and enhancing HS tolerance. Four significant steps could be taken to utilize these techniques, (I) Identifying HS-responsive genes, (II) Targeted gene editing, i.e., to protect photosynthesis (RuBisCo, RCA, D1, IF, HYR, and TT3.1–TT3.2), improving stomatal closure and cuticle deposition to prevent water loss (MDHAR4, HTAS, DST, and the TT2-SCT1-WR2 regulatory pathways), optimizing the heading/flowering stage (pathways MADS51/qHd1–Ehd1–RFT1/Hd3a and Ghd7–Ehd1–RFT1/Hd3a or specific genes such as EG1 and DFOT1), enhancing fertility (through increased osmoproctants, balanced ROS homeostasis, stress responsive metabolites) and maintaining normal endosperm development under HS to reduce grain chalkiness and enhance grain weight and quality (ONAC127/129, Fie1, MADS87, MADS57-Wx, DOF17–WCR1–MT2b (Metallothionein 2b), bZIP60, bZIP58–Amy1C/3A/3D, TT1, Hsp70cp-2, and ZmPGD3 homologs) through CRISPR-Cas9, ZFNs, TALENs etc. (III) Incorporation of edited gene into the plant cells, and (IV) Confirmation of genome editing in heat tolerant rice plant. **(D)** Data-driven approaches: Precise, accurate, reliable, and reproducible phenotyping is needed to identify the most important traits and promising donors with superior characteristics for use in HS tolerance breeding programs in rice. Mobile and stationary platforms are two primary categories in high-throughput phenotyping platforms. Mobile platforms include field-based platforms (satellite imagery, UAV imaging, and ground-based imaging through PhenoMobile) and laboratory-based platforms (MVS-Pheno). Stationary platforms include facilities such as the Nanaji Deshmukh Plant Phenomics Centre (NDPPC), New Delhi. Imaging techniques are an essential part of high-throughput phenotyping (HTP). Magnetic Resonance Imaging (MRI), X-ray/CT (X-ray Computed Tomography), RGB (Visible light), Infrared thermal (IR) imaging, fluorescence imaging, and LiDAR (Light Detection and Ranging). These HTP facilities can transform HS phenotyping and screening with high spatial and temporal resolution. By combining machine learning (ML) and deep learning (DL) in HTP, we may be able to use deep neural networks to evaluate big data sets and images with more precision and accuracy.

Improving yield under stress conditions through direct selection is constrained by the low heritability and intricate nature of the QTLs that govern them. Marker-assisted selection (MAS) has proven effective in precisely transferring genes from wild germplasm while minimizing linkage drag, as it can integrate modern classical genetics, bioinformatics, and conventional biotechnology effectively. Techniques such as association mapping and bi-parental mapping can elucidate the connection between phenotypic variation and genetic polymorphism, facilitating the mapping of relevant genomic regions (Visakh et al., 2024). After mapping QTL regions associated with thermotolerance, these regions can be incorporated into superior genetic backgrounds employing MAS in breeding programs. Utilizing marker-assisted backcrossing (MABC), a few lines have been developed for HS tolerance. For example, Lang et al. (2015) applied MABC using six markers to enhance heat tolerance in rice varieties in Vietnam. They crossed heat-tolerant germplasms (N22 and Dular) with five high-yielding *indica* cultivars, resulting in the development of four heat-tolerant lines by the BC_4_F_2_ generation. Ye et al. (2022) introgressed two QTLs, namely *qEMF3* and *qHTSF4.1*, for early morning flowering and thermotolerance, respectively, into the background of IR64, and a QTL pyramiding line IR64+qHTSF4.1+qEMF3 was developed using MAS. In another effort in marker-assisted pedigree breeding by Withanawasam et al. (2022), they developed PL457 and PL130 with heat-tolerant QTLs *qSSP_F_10* and *qHT6*. These lines showed 85.02% and 61.55% yield advantages, respectively over IR64. Similarly, Vivitha et al. (2017) utilized MABC to introduce QTLs (*qHTSF1.1* and *qHTSF4.1*) into Improved White Ponni lines, effectively enhancing thermotolerance. (**Figure 7A**).

### 11.3 Genome editing and transgenic approach

Genome editing technologies in crop plants are advancing rapidly, allowing for targeted mutations with exceptional specificity and accuracy. Unlike genetic engineering, genome editing doesn’t involve the integration of foreign DNA into plants, making the final product indistinguishable from the parent plants without changing the overall stability of the genome. Genome editing tools such as zinc finger nucleases (ZFN), meganucleases, transcription activator-like effector nucleases (TALEN), and CRISPR/Cas system offer promising avenues for creating improved plant varieties by adding desirable traits or removing undesirable ones (Noman et al., 2016). Among these, CRISPR system stands out for its precision in modifying DNA at specific locations. It is being extensively utilized for the rapid, easy, and efficient alteration of genes to enhance HS in rice and to understand the function of specific genes by creating gene knockout mutants (Biswal et al., 2019; Tingting et al., 2023).

Qu et al. (2021) co-overexpressed RuBisCo and RuBisCo activase in rice plants and analyzed the photosynthesis and biomass at 25°C and 40°C. Plants showed a higher CO_2_ assimilation rate at 40°C, which resulted in 26% higher dry weight than the wild types. To stimulate β-glucuronidase (GUS) gene expression in rice, promoters of the three highly heat-inducible genes *OsHsfB2cp* (a HSF), *PM19p* (a stress responsive promoter), and *Hsp90p* were employed. The transgenic rice panicles and flag leaves validated high heat-induced GUS activities, mild drought-induced activities through GUS gene expression and histochemical staining. While *OsHsfB2cp* and *PM19p* showed significantly higher activities in panicles under HS, the three promoters displayed comparable high activity levels in rice leaves (Rerksiri et al., 2013). *OsDHSRP1* (an E3-ubiquitin ligase) regulates plant abiotic stress tolerance via the Ub/26S proteasome system, and its transcripts are highly expressed in heat and drought conditions. The Arabidopsis showed hypersensitivity to heat by reducing its germination rates and root length when *OsDHSRP1 was* overexpressed. It suggests that *OsDHSRP1* E3 ligase acts as a negative regulator, and the degradation of its substrate proteins via ubiquitination has an essential role in the modulation of HS response via an ABA-independent pathway (Kim et al., 2020). When *ERECTA* (receptor-like kinase)-overexpressing plants were subjected to HT (42°C Day/35°C night) for 10 days at the reproductive stage, most leaves and tillers of retained their green color and survived as compared control lines. Additionally, the seed-setting rate of *ERECTA*-overexpressing plants was 55%–70% higher than the control line following HTS (Shen et al., 2015) (**Table 4**). When a zinc finger protein gene, *OsZFP350*, was upregulated, transgenic rice plants showed enhanced primary root length, adventitious and lateral roots, and significantly increased germination rate (Kang et al., 2019).

**Table 4:** HS-tolerant transgenic rice: Genes and their functions.

Gene	Source	Encoding protein	Function	Regulation*	Reference
*Athsp101*	*Arabidopsis*	Hsp 101	Better growth performance in the recovery phase following the stress	+	Katiyar-Agarwal et al. (2003)
*sHSP17.7*	*O. sativa*	Class I cytosolic small HSP	Seedlings exhibited increased thermotolerance and tolerance to ultraviolet B damage	+	Murakami et al. (2004)
*SBPase*	*O. sativa*	Sedoheptulose-1,7-bisphosphatase	Accumulated SBPase in chloroplast, maintain RuBisCo activation by preventing RuBisCo activase sequestration thylakoid membranes from soluble stroma fraction, thereby increasing CO_2_ assimilation and enhancing tolerance to HT	+	Feng et al. (2007)
*OsHsfA2e*	*O. sativa*	Heat stress TFs	Increased HS tolerance thermotolerance in *Arabidopsis*	+	Yokotani et al. (2008)
*OsWRKY11*	*O. sativa*	a TF with the WRKY domain	Slower leaf-wilting and a less-impaired survival of plant’s green tissues	+	Wu et al. (2009)
*mtHsp70*	*O. sativa*	Mitochondrial heat shock protein 70	Inhibited heat- and H_2_O_2_-induced programmed cell death in rice protoplasts, evidenced by higher cell viability, decreased DNA laddering, and chromatin condensation.	+	Qi et al. (2011)
*OsMYB55* (transciption factor)	*O. sativa*	MYB proteins	Enhances amino acid metabolic pathways and increases total amino acid content under HTS	+	El-kereamy et al. (2012)
*OsHCI1 (Oryza sativa heat and cold-induced 1)*	*O. sativa*	RING finger protein	During HS, regulates nuclear-cytoplasmic trafficking of nuclear substrate proteins through monoubiquitination and drives an inactivation device for the nuclear proteins	+	Lim et al. (2013)
*MSD1*	*Arabidopsis*	Superoxide dismutase	Up-regulated reactive oxygen scavenging, chaperone, and quality control systems in rice grains	+	Shiraya et al. (2015)
*ER (ERECTA)*	*Arabidopsis*	*receptor-like kinase*	Enhance heat tolerance without affecting water loss	+	Shen et al. (2015)
*DPB3-1*	*Arabidopsis*	transcriptional regulator DNA polymerase II subunit B3-1	Positive regulator of Dehydration-responsive element binding protein 2A (DREB2A); many HS-inducible genes were up-regulated at vegetative and reproductive stages	+	Sato et al. (2016)
*TOGR1 (Thermotolerant Growth Required1)*	*O. sativa*	DEAD-box RNA helicase	Modulates a normal rRNA homeostasis at HT	+	Wang et al. (2016)
*OsHTAS*	*O. sativa*	a ubiquitin E3 ligase	Regulating H_2_O_2_ accumulation in shoots, altering leaf stomatal aperture, and enhanced ABA biosynthesis in seedlings	+	Liu et al. (2016)
*OsOPT10 (O.sativa Oligopeptide Transporter 10)*	*O. sativa*	oligopeptide transporter	Increased heat tolerance by regulating electrolyte leakage, soluble sugars, and proline content	+	Jeong et al. (2017)
*OsbZIP46CA1*	*O. sativa*	bZIP transcription factor	Enhanced thermotolerance when co-expressed with a protein kinase (*SAPK6*)	+	Chang et al. (2017)
*OsMDHAR4*	*O. sativa*	Monodehydroascorbate reductase	Supersession enhanced thermotolerance by facilitating H_2_O_2_-induced regulation of stomatal closure	_	Liu et al. (2018)
*Sus3*	*O. sativa*	Sucrose synthase	Catalyzes first step of starch synthesis, conversion of sucrose and uridine diphosphate (UDP) to fructose and UDP-glucose: HT tolerance during ripening	+	Takehara et al. (2018)
*Rca*	*O. australiensis*	Thermostable variants of the photosynthesis heat-labile protein RuBisCo activase	Enhanced carbohydrate accumulation and storage	+	Scafaro et al. (2018)
*OsMADS7*	*O. sativa*	TF	Suppression stabilizes amylose content under HTS but results in low spikelet fertility; it could be overcome by endosperm-specific suppression	_	Zhang et al. (2018)
*OsIF*	*O. sativa*	Intermediate filaments	Stabilizes photosynthesis by maintaining the ultrastructure of the chloroplast; survival and yield increase	+	Soda et al. (2018)
*Rab7*	*O. sativa*	Small GTP-binding proteins	Enhance thermotolerance by influencing osmolytes, antioxidants, and expression of stress-responsive gene	+	El-Esawi and Alayafi (2019)
*OsHBP1b*	*O. sativa*	Histone gene binding protein	Transgenics had better roots, large cortical cells, and a good amount of callose accumulation; improved shoot growth, enhanced photosynthesis, and elevated antioxidant enzyme activity	+	Das et al. (2019)
*PDI*	*Methanothermobacter thermautotrophicus*	MTH1745 (disulfide isomerase-like protein)	Increased proline content, superoxide dismutase, and peroxidase activities enhanced thermotolerance in seedlings	+	Wang et al. (2019)
*OsFBN1*	*O. sativa*	plastid-lipid-associated (PAPs) protein	Overexpressing increased the tiller number but decreased the panicle length, grain-filling, and jasmonate content	_	Li et al. (2019)
*OsHIRP1 (Oryza sativa heat-induced RING finger protein 1)*	*O. sativa*	E3 ligase	High germination and survival rates	+	Kim et al. (2019)
*OsUBP21*	*O. sativa*	ubiquitin-specific protease	Knocking the expression down or out increases the thermotolerance	_	Zhou et al. (2019)
*OsRGB1*	*O. sativa*	Rice beta subunit (RGB1) of the G-protein	Lower electrolyte leakage and malondialdehyde production while showing higher levels of chlorophyll, higher germination rate, root length, shoot length, and plant height	+	Biswas et al. (2019)
*psbA*	*Arabidopsis*	D1 Protein	Involved in photosystem II repair; enhances heat tolerance by maintaining D1 protein levels	+	Chen et al. (2020)
*SLG1(Slender Guy 1)*	*O. sativa*	tRNA 2- thiolation protein 2 (RCTU2)	Increased the thiolated tRNA level and enhanced the thermotolerance at seedling and reproductive stages.	+	Xu et al. (2020)
*AtPLC9*	*Arabidopsis*	Phosphoinositide-specific phospholipase C	Regulates auxin levels in the vegetative and floral organs to influence male and female gametophytes organ formation	+	Liu et al. (2020)
*OsNAC006*	*O. sativa*	TF	Knockout showed heat sensitivity; genes associated with stimulus response, oxidoreductase activity, cofactor binding, and membrane-related pathways	+	Wang et al. (2020)
*OsERF115/AP2EREBP110*	*O. sativa*	TF	Enhanced proline level and upregulation of proline biosynthesis *P5CS1* gene	+	Park et al. (2021)
*ONAC127, ONAC129*	*O. sativa*	TF	Overexpressed plants exhibited poor grain filling and shrunken grains	_	Ren et al. (2021)
*PSL50 (PREMATURE SENESCENCE LEAF 50)*	*O. sativa*	Clathrin-associated adaptor protein complex 1 medium subunit μ1 (AP1M1)	Enhanced thermotolerance by modulating H_2_O_2_ signaling	+	He et al. (2021)
*Rca1β*	*T. aestivum*	RuBisCo activase B	Better photosynthate energy partitioning under HS; reduction in the non-photochemical fluorescence quenching of the photosynthetic machinery	+	Chaudhary et al. (2021)
*F3H*	*O. sativa*	Flavanol 3-hydroxylase	Higher biosynthesis of kaempferol and quercetin	+	Jan et al. (2021)
*ZmHsf11*	*Z. mays*	HSFs	Overexpression reduced the survival rate, accumulated more H_2_O_2_, increased cell death, and decreased proline content	_	Qin et al. (2022)
*HTG3a (Heat-Tolerance Gene on Chromosome 3)*	*O. sativa*	Heat-shock factor	Enhanced thermotolerance at vegetative and reproductive stages by regulating *JAZs* and other heat-responsive genes	+	Wu et al. (2022)
*HTH5*	*O. rufipogon*	pyridoxal phosphate homeostasis protein (PLPHP)	ROS scavenging via elevated heat-induced pyridoxal 5’-phosphate (PLP) content, improved seed-setting during heading	+	Cao et al. (2022)
*TT2(THERMOTOLERANCE 2)*	*o.glaberrima*	encoding a Gγ subunit	Loss of function led to higher wax retention at HT and enhanced thermotolerance	_	Kan et al. (2022)
*OsNRT2.3*	*O. sativa*	Encodes a high-affinity nitrate transporter	Sustain high productivity and efficinet nitrogen use under HT	+	Zhang et al. (2022)
*OsNCED1*	*O. sativa*	9-cis-epoxycarotenoid dioxygenase (NCED)	Increase antioxidant capacity; enhances thermotolerance at the heading and flowering stage	+	Zhou et al. (2022)
*OsSGS3a (O. sativa SUPPRESSOR OF GENE SILENCING 3a)*	*O. sativa*	SGS3 protein	Modulates biogenesis of *trans*-acting small interfering RNA (tasiRNA) modulating *AUXIN RESPONSE FACTORS* (*ARFs*)	+	Gu et al. (2023)
*OsHis1.1*	*O. sativa*	Histone H1	Inhibiting heat responsive genes, overexpressing lines exhibited lower POD activity, chlorophyll, and proline contents; suffered severe oxidative stress and cell damage	_	Wan et al. (2023)
*OsHSP 17.9*	*O. sativa*	HSP	Enhanced functioning of antioxidant enzymes	+	Do et al. (2023)
*OsGRP3/OsGRP162*	*O. sativa*	glycine-rich RNA-binding proteins	Thermotolerance in a diurnal manner, especially at night time	+	Yang et al. (2024)

*In regulation column, “+” and “-” indicate upregulation and downregulation of genes, respectively.

Silencing of CI-sHsps (Class I small HSPs) by RNAi negatively affected rice seedlings’ seed germination process and HS response. The seed length was reduced, the seed germination process was delayed, and seed thermotolerance was negatively affected compared to non-transgenic seeds (Sarkar et al., 2019). Eliminating ABA receptor (*PYL1/4/6*) encoding genes via CRISPR/Cas9 has conferred substantial thermotolerance in rice (Miao et al., 2018). Rice knockout mutants of *OsHSP60-3B* demonstrated normal fertility under favorable temperatures but experienced fertility reduction with rising temperatures. Pollen from mutant *oshsp60-3b* shows decreased starch accumulation and viability, while anthers produce enhanced levels of ROS, resulting in cell death. Further exploration of this gene and its implications for HS response is warranted (Lin et al., 2023).

Genome editing provides a fast approach for developing thermotolerant rice varieties compared to conventional methods by precisely targeting specific genes, thus enabling fine-tuning tolerance. If these advanced approaches are suitably amalgamated with techniques such as speed breeding and genome-wide association studies (GWAS), it will revolutionize the efforts for developing HS-resilient rice varieties (**Figure 7C**).

### 11.4 Omics approaches for developing heat-tolerant rice

Large-scale, high-throughput approaches such as genomics, transcriptomics, proteomics, metabolomics, epigenomics, hormonomics, ionomics, and phenomics are central to omics technology. Omics in rice research has deepened the understanding of complex molecular responses, differential gene expression, regulatory pathways, comprehensive genome-wide insights into gene structures, their functions, interconnected regulatory networks, metabolic and biochemical processes they participate in, thereby helping to elucidate how gene networks interact with complex stress resilient traits with potential applications in crop improvement for thermotolerance (Iqbal et al., 2021; Pandian et al., 2020; Singh et al., 2024; Varshney et al., 2018; Zhang et al., 2019).

Proteomic studies have shown stage specific patterns of differentially expressed proteins at various developmental stages under HTS. These proteins are primarily associated with biosynthesis, energy and metabolic processes, redox balance, photosynthesis, and signaling pathways, and contribute to short term protective response that enhance thermotolerance (Guo et al., 2024). In various extensive studies, heat-responsive QTLs and genes have been identified and cloned, such as by Huang et al. (2023) and Nguyen et al. (2022). Li et al. (2023) identified a major locus *qHT7* through GWAS and further transcriptome analysis revealed candidate gene *LOC_Os07g48710 (OsVQ30*, a transcriptional regulatory factor*)* within this locus. Liu et al. (2021) studied regulatory effects of HT on grain development and material accumulation pathways. Proteomic findings revealed a total of 840 differentially expressed proteins during the grain filling process. Proteins such as PPROL 14E, PSB28, granule-bound starch synthase I were upregulated.

Transcriptome of a rice hybrid showed upregulation of genes involved in responses to stimuli, cell communication, and metabolic and TF activities while down regulated genes were enriched in photosynthesis and signal transduction (Wang et al., 2020). Heat-tolerant genotype, SDWG005, maintained a steady state balance of metabolic processes, associated with reprogramming cellular activities and had a critical role in preserving heat tolerance (Cai et al., 2020) (**Table 5**). HS changes phosphorylation dynamically and alters its pattern in enzymes related to starch biosynthesis (Pang et al., 2021). Roots of heat tolerant cultivar mainly activated pathways involving phenylalanine/phenylpropanoid, aromatic amino acid, lysine degradation, branched chain amino acids, glycerophospholipids, and alkaloids while heat susceptible cultivar emphasized nitrogen-related and antioxidant pathways under HTS in a metabolomic study by Ogawa et al. (2025) (**Table 5**). Targeted metabolomic studies are essential to elucidate inter-organ communication and hormonal crosstalk in rice during HTS (Prerostova et al., 2022).

**Table 5:** Recent studies on HS tolerance in rice using different omics technologies.

Omics technology	Rice genotype(s)	Tissue(s)	Key findings	Reference
Transcriptomics and Proteomics (2D-PAGE and MS/MS)*	Hybrid rice II YOU 838 (II8) and its parents Fu Hui 838 (F8) and II-32A (II3)	Flag leaves (flowering stage)	Hybrid showed greater heat tolerance. Hsp70, CPN60 (chaperonin), bHLH96, calmodulin-binding transcription activator were among the unique differentially abundant proteins. HSF-Hsp regulatory network plays a central role. Overexpression of HSF and HSP family genes contribute to heterosis for heat tolerance	Wang et al. (2020)
Metabolomics (GC-MS)*	CT9993-5-10-1M, IR123, IR62266-42-6-2, IR64, IR72, M202, Moroberekan, Taipei309	Flag leaves and panicles (flowerin stage)	Impaired glycolysis and higher respiration driven carbon loss. Polyols (arabitol, erythritol) increased under HNT.	Schaarschmidt et al. (2020)
Metabolomics (picoPPESI-MS)	N22 (heat-tolerant) and Koshihikari heat-sensitive)	Single mature pollen grains (ungerminated)	N22 had higher phosphatidylinositol (a precursor for phosphoinositide signaling) accumulation.	Wada et al. (2020)
Transcriptomics	SDWG005 (heat-tolerant African landrace) and 9311 (heat-sensitive restorer lines)	Anthers (anthesis stage)	Anther specific gene, *OsACT* (agmatine-coumarin-acyltransferase) showed differential expression and promoter polymorphism linked to thermotolerance. SDWG005 maintained stable structure under HS	Liu et al. (2020)
Phosphoproteomic	9311 and Guangluai4 (GLA4)	Developing endosperm	HTS altered phosphorylation patterns, especially in starch biosynthesis enzymes (AGPase, GBSSI, SSIIIa, BEI, BEIIb). Consensus motif ([sP], [LxRxxs], [Rxxxs], [tP]) linked to CDPK kinases activated by HS.	Pang et al. (2021)
Transcriptomics	Kitaake	Developing seeds (early post-fertilization stage)	Rice seeds are most sensitive during 0–2 days after fertilization. Inositol-requiring enzyme 1(IRE1)-mediated ER stress pathway and jasmonic acid pathway are activated first during HS. ER stress accelerates endosperm cellularization by upregulating genes like OsFIE1 and *OsbZIP76*	Sandhu et al. (2021)
Transcriptomics	Annapurna (heat-tolerant) and IR 64 (heat-sensitive)	Whole seedlings	Annapurna showed unique perturbation of auxin and ABA signaling pathways involving genes OsIAA13, OsIAA20 (transcriptional repressor in auxin signaling), ILL8, OsbZIP12, OsPP2C51 (encodes protein phosphatase 2C), OsDi19-1 (TF) and OsHOX 24 (homeobox TF)	Sharma et al. (2021)
Transcriptomics and metabolomics (UPLC-MS/MS)*	Nipponbare and OsPHYB knockout mutants	Developing grains (endosperm)	Knockout of OsPHYB (rice phytochrome B) increased grain size and chalkiness, and altered nutrient composition. OSPHYB regulated grain traits via carbon metabolism, hormone signaling, cell cycle and antioxidant pathways	Li et al. (2022)
Transcriptomics	T11 (heat-tolerant) and T15 (heat-sensitive)	Leaves (seedling stage)	Early activation of MAPK siganling pathway in T11. Involved pathways: protein processing in ER, hormone signalling, lipid metabolism. Reported candidate genes *LOC_Os05g23140* (member of Hsp20/α crystallin group) and *LOC_Os05g11140* (encodes tyrosine kinase) to be HS responsive	Cai et al. (2023)
Transcriptomics	T2 (Jinxibai, heat-tolerant) and T21 (Taizhongxianuan2hao, heat-sensitive)	Leaves (seedling stage)	Pathways regulating protein processing in ER, plant hormones signal transduction, MAPK signaling, and carbon metabolism are important for thermotolerance. T2 had higher proline accumulation and better antioxidant enzyme activity. *Os05g45410/OsSPL7/OsHsf4d* balances ROS under HS.	He et al. (2023)
Epigemomics (ATAC-Seq)* and Transcritpmics	Nipponbare	Leaves (seedling stage)	Identified three key heat responsive TFs: *OsbZIP14* (a nuclear TF with transcriptional activationability), *OsMYB2*, and *OsHSF7*.	Qiu et al. (2023)
Proteomics	Huang Huazhan (HZ) (heat-tolerant) andYangdao6(YD) (heat-susceptible)	Anthers	Higher antioxidant enzyme activities, proline and sugar in HZ. Phenylpropanoid biosynthesis, ubiquitin mediated proteolysis, carbohydrate, and thiamine metabolism, and protein processing in ER. Higher expression of candidate genes *LOC_Os08g07010* and *LOC_Os08g07440* in HZ	Guo et al. (2024)
Metabolomics	15 genotypes (14 aromatic and 1 no-aromatic:N22)	Rice grains (grain filling stage)	No 2-acetyl-1-pyrroline (2-AP) accumulation detection under HS. L-proline levels increased significantly during HT but its conversion to 2-AP was impaired due to *BADH2* down regulation	Mishra et al. (2024)
Transcriptomics and metabolomics (UHPLC-MS/MS)*	R28, R18 and Q3B	Young panicles, unopened florets, and developing grains	Identified stage specific key genes; Transcriptomics - flavonoid 3-hydroxylase (booting), mannan endo-1.4-beta-mannosidase and hexokinase (flowering). Metabolomics – heat tolerance linked to metabolites such as kaempferol and rhoifolin (booting), arachidonic acid and eicosatetraenoic acid (flowering), and vindolines (grain filling) Integrated analysis revealed three important pathways: flavonoid biosynthesis (booting), fructose/mannose metabolism (flowering), and α-linolenic acid metabolism (grain filling)	Guan et al. (2025)
Transcriptomics and metabolomics (UPLC-MS/MS)*	DY80 (Heat-tolerant accession of Dongxiang wild rice) and R974 (heat-sensitive)	Leaves (seedling stage)	Transcriptomics: 1817 DEGs unique to DY80: pathways involved include unfolded protein binding (upregulated), chlorophyl biosynthesis, cysteine and methionine metabolism, photosystem I and II (downregulated). Metabolomics: unique metabolites in DY80 were malic acid, stearic acid, and L-threonine linked to thermotolerance	Zhang et al. (2025)
Transcriptomics	Four weedy rice accession: heat-tolerant – MU235, MU244; heat-susceptible – MU005, MU251, and Controls: MR219, MU201 (wild *O. rufipogon*)	Whole seedlings (3–4 leaf stage)	Upregulation of genes in ER protein processing and HSPs in heat tolerant genotypes. Heat susceptible genotypes showed downregulation of oxidative phosphorylation pathway genes. Candidate genes for rice thermotolerance included – *GA2OX7, OsLFNR2, OsPCNA, OsHSC70, OsBiP4, HSFA2B, OsHSFB2B, OsPDS*	Sarker et al. (2025)
Proteomics (LC/MS-MS)*	N22	Spikelets	Upregulated proteins linked to translation, ribosome structure, cytoplasm, and metabolic pathways, suggesting enhanced energy generation and protein repair	Ju et al. (2025)
Transcriptomics	BR-IRGA 409 (moderately heat-tolerant) and IRGA 428 (heat-susceptible)	Flag leaves and spikelets	Upregulation of heat responsive genes (HSFs, HSPs, and peptidyl-prolyl isomerase FK506-binding proteins), Increased expression of mitochondrial electron transport chain genes indicating enhanced ATP production and energy homeostasis.	Correia et al. (2025)
Metabolomics (CE-MS)*	Fusaotome (heat-tolerant) and Akitakomachi (heat-sensitive)	Panicles and roots (heading stage)	Activation of tryptophan, nicotinate/nicotinamide, arginine/proline, glycolysis/TCA cycle, vitamin B6 pathways in Fusaotome. Enrichment of secondary defense pathways in Akitakomachi.	Ogawa et al. (2025)
Metabolomics and (LC/MS-MS), Transcriptomics	ZH11 (wild type), OsDUGT1 overexpression lines (OE19, OE21), and Osdugt1 knockout mutants (ko18, ko78)	Leaves (seedling stage)	*OsDUGT1* is heat inducible, its overexpression reduces ROS, MDA, and ion leakage. It glycosylates flavonoids.	Dong et al. (2025)
Transcriptomics	93-11 (*indica*) and ZH11 (*japonica*)	Root (seedling stage)	Identified *OsMAPK3* (mitogen-activated protein kinase) as a novel hub gene	Deng et al. (2025)

*Ultra-High-Performance Liquid Chromatography-Tandem Mass Spectrometry (UHPLC-MS/MS), Ultra-Performance Liquid Chromatography-Tandem Mass Spectrometry (UPLC-MS/MS), Liquid Chromatography-Mass Spectrometry (LC/MS-MS), Capillary Electrophoresis-Mass Spectrometry (CE-MS), Two-Dimensional Polyacrylamide Gel Electrophoresis (2D-PAGE), Tandem Mass Spectrometry (MS/MS), Assay for Transposase Accessible Chromatin with High-Throughput Sequencing (ATAC-Seq), picolitre pressure-probe electrospray ionization mass spectrometry (picoPPESI-MS), Gas-Chromatography-Mass Spectrometry (GC-MS).

HS alters various aspects of chromosomes organization, including shifts between A and B compartments, enlarged topologically associated domains, and reduced short range chromatin interactions. These structural modifications correlate with the changes in chromatin accessibility and gene expression (Liang et al., 2021). HS significantly alters splicing patterns in rice. Two notable candidate genes, *LOC_Os03g16460* (encoding an uncharacterized protein) and *LOC_Os05g07050* (encodes pre-mRNA splicing factor 8) were linked to alternate splicing and intron retention (IR) type of alternate splicing being more common under HS (Yang et al., 2022). A rice specific DOF transcription factor, *OsDOF27* (an intrinsically disordered protein) is highly regulated under HS. Its promoter region in enriched with abiotic stress and phytohormone-responsive cis-elements (Gandass et al., 2022). Ubiquitination, a major post-translational modification, contributes critically to thermotolerance. Ying et al. (2023) identified 488 ubiquitination sites in 246 proteins in rice endosperm during HTS in a ubiquitomic study. Ubiquitination likely contributes to decreased abundance of starch synthesis enzymes, explaining reduced amylose content under HS.

Studies using omics technologies in rice have revealed significant genetic and molecular factors that help to understand the intricate mechanism of HS tolerance (**Table 5**). Developments in computational biology and bioinformatics have significantly improved the analysis and interpretation of proteomic data. Machine learning tools for network analysis and algorithms determine the critical regulatory proteins and their interaction, shedding light on the intricate regulatory networks. These holistic approaches has resulted in identifying possible protein targets for breeding and genetic engineering to create rice cultivars that can withstand HT (Jhan et al., 2023; Singh et al., 2024) (**Figure 7B**).

### 11.5 Harnessing plant-microbe interactions to improve thermotolerance in rice

Rice plants’ microbiome consists of diverse microorganisms (bacteria, archaea, fungi, and viruses) present both inside and outside of their tissues, in the endosphere and ectosphere, respectively (Singh et al., 2022; Aswini, 2023; Zhao et al., 2024). Beneficial micro-organisms can help to reprogram the plant epitranscriptome to enhance thermotolerance (Shekhawat et al., 2022). Waqas et al. (2015) reported that the endophytic fungus *Paecilomyces formosus* LWL1 produces phytohormones and organic acids, which enhanced the HS tolerance of a *japonica* variety, Dongjin. *Bacillus amyloliquefaciens* NBRI-SN13 (SN13) ameliorated various abiotic stresses, including HS in a rice variety Saryu-52 (Tiwari et al., 2017). In both the rhizosphere and endosphere, a significant decline in microbial abundance has been observed in the IR64 (temperature sensitive) cultivar compared to the Huanghazou (temperature resistant) cultivar (Munir et al., 2023). Rice plants inoculated with *Brevibacterium linens* RS16, which produces 1-aminocyclopropane-1-carboxylate (ACC) deaminase, had enhanced tolerance against UV-b radiation, and 40°C of temperature because of decreased ethylene emissions, increased plant biomass and photosynthesis, and restricted DNA damage (Choi et al., 2022).

Microorganisms enhance HS tolerance by the production of antioxidants, plant growth hormones, bioactive compounds, detoxifying harmful compounds, sequestration of ROS and other free radicals, and releasing protective substances to withstand desiccation (Singh et al., 2023; Hosseiniyan Khatibi et al., 2024). So, understanding and deciphering the mechanics and interplay of the phyto-microbiome in rice with HS tolerance using the potential of multi-omics, e.g., metagenomics and meta-transcriptomics approaches, holds great promise to uncover the complex microbial network involved in stress signaling and development of stress tolerance (**Figure 7B**).

## 12 Integrating high-throughput phenotyping and artificial intelligence for HS tolerance breeding

The complexities imposed by HTS at the cellular level are further compounded at the physiological level by interactions between phytohormones and gene-regulatory proteins. This complexity is further heightened with interactions between different temperatures (e.g., high day and night temperatures) and other abiotic stresses, such as drought. Thus, accurately and precisely measuring traits impacted by HTS necessitates careful observation and interpretation, often in real-time. High throughput phenotyping (HTP), which uses imaging techniques, including fluorescence, visible range, near-infrared, laser, thermal, and hyperspectral imaging, has enabled measuring traits with extreme precision and at a high temporal and spatial scales in a non-destructive manner on a large scale to characterize crop response to HT (Bahuguna and Jagadish, 2015; Kundu et al., 2024). In addition to that, conventional plant phenotyping is time-consuming, labor-intensive, and expensive. HTP can be further improved by integrating artificial intelligence (AI) techniques for collecting reliable data to identify promising traits and genotypes for faster genetic gain and increased heritability. Deep phenotyping enables observing changes in tissue composition (e.g., proteins, lipids, carbohydrates, and metabolites) *in vivo* (Kundu et al., 2024). High frequency, high resolution imaging using a unmanned aerial vehicle (UAV) quantified dynamic drought responses of a rice population under field conditions using deep convolutional neural networks (DCNNs) together with canopy heights models. Traits such as UAV-based leaf rolling score, plant water content, and drought resistant index by UAV were measured. Genome wide association analysis identified 111 significant loci associated with three dynamic traits (Jiang et al., 2021). Convolutional neural networks (CNNs) and Gradient-weighted Class Activation Mapping (Grad-CAM) based approach was used to detect chalkiness in rice grains (Wang et al., 2022). Payman et al. (2018) used computer vision system to assess rice appearance qualities such as whiteness and chalkiness. Thermal stressed-induced spikelet sterility was investigated using a crop-model-assisted GWAS approach. Parameters provided RIDEV model was able to give more and strong QTLs then traits taken by observation (Dingkuhn et al., 2017). A high-throughput chlorophyll fluorescence platform enables rapid screening of photosynthetic heat tolerance in rice, revealing highly heritable PSII heat tolerant traits, substantial genetic variation across diverse germplasm, and 133 candidate genes underlying PSII thermotolerance through GWAS (Robson et al., 2023).

Combining HTP, AI, deep learning, and integrated data networks can help developing large repositories of genotyping data, producing big and accurate phenotypic data sets (Gill et al., 2022; Khatibi and Ali, 2024), accelerating the pace of developing stress resilience and discovering new traits and genes for HS tolerance as shown in **Figure 7D**.

## 13 Conclusion

Increasing efforts to develop heat-tolerant varieties become critical for food security amid climate change (Kan et al., 2023). Plenty of information is available about the physiological and metabolic traits that govern heat tolerance, but the underlying complex mechanisms are still unknown. Construction of high-density QTL maps for heat tolerance has provided a strong foundation. However, effective utilization of genetic resources, which have already been identified in breeding programs, remains a challenge. Approaches such as transgenics and advanced molecular breeding play a critical role in determining rice’s responsible genes and complex pathways governing HS tolerance. Genome editing techniques such as CRISPR/Cas9 and RNAi could be promising techniques. Advances in sequencing and high-throughput omics technologies have led to the generation of a vast array of omics data. Genomic regions governing heat tolerance can be identified by genomics and genetic mapping. Integrative studies of different disciplines such as structural genomics, proteomics, transcriptomics, and metabolomics with rice physiology and breeding would accelerate the efforts to identify key proteins, novel marker genes, and metabolic pathways to elucidate molecular mechanisms of thermotolerance and develop heat-tolerant rice varieties. Under stress conditions, various genes are expressed differentially, which is regulated by several processes, including N-tail modifications, histone variants, and DNA methylation. Comprehensive research of epigenetic regulatory mechanisms under HTS, particularly reproductive and grain-filling stages, needs to be extended and investigated in field conditions, as rice may encounter drought along with HS (Begcy and Dresselhaus, 2018). Incorporation of various HTP technologies integrated with AI tools assists in precise, detailed, deep, and large-scale phenotyping, considering the complexities imposed by HS. Microbial communities show greater resilience towards changes in the environment. Multi-omics approaches could play a key role in deciphering rice’s HS tolerance mechanism governed by plant and microbiome interactions. This integration of different methods in MAS and genomic selection will increase efficiency and accuracy in enhancing heat tolerance through breeding programs.
